# Cannabinoids for treating psychiatric disorders in youth: a systematic review of randomized controlled trials

**DOI:** 10.1186/s13034-024-00846-5

**Published:** 2024-12-18

**Authors:** Patrick Köck, Andrzej Badek, Maximilian Meyer, Arndt-Lukas Klaassen, Marc Walter, Jochen Kindler

**Affiliations:** 1 Department of Psychosomatics and Psychotherapy, Clinic Barmelweid, Barmelweid, Switzerland; 2 University Hospital of Child and Adolescent Psychiatry and Psychotherapy, University of Bern, Bern, Switzerland; 3 Department of Psychiatry, University Clinics of Psychiatry Basel, University of Basel, Basel, Switzerland; 4 Department of Anesthesiology & Pain Medicine, Bern University Hospital, Inselspital, University of Bern, Bern, Switzerland; 5 Clinic of Psychiatry and Psychotherapy, Psychiatric Services Aargau, Windisch, Switzerland; 6 Child and Adolescent Psychiatry, Psychiatry Baselland, Liestal, Switzerland

**Keywords:** Cannabinoid, Cannabidiol, Youth, Mental disorder, Cannabis therapy, Autism spectrum disorder, Psychotic disorders

## Abstract

**Background:**

Cannabinoids have been of increasing interest mainly due to their putative efficacy in a wide array of psychiatric, psychosomatic, and neurological conditions.

**Aims:**

This systematic review aims to synthesize results from randomized placebo-controlled trials regarding the efficacy and the dosage of cannabinoids as therapeutics in psychiatric disorders in children, adolescents, and young adults.

**Methods:**

All publications up to June 30th, 2024, were included from PubMed and Embase. Eligibility criteria in accordance with the PRISMA-guidelines was applied. RCTs providing pre- and post-treatment parameters on cannabinoid therapies for mental disorders in comparison to controls in an age range from 0 to 25 years were included. Effect sizes were calculated as Hedges’ g for primary outcomes, and a multilevel random-effects meta-analysis was conducted to account for dependent outcomes from same study populations.

**Results:**

We identified 7603 records, of which 8 independent clinical trials (reported in 9 publications) met the pre-established eligibility criteria, comprising 474 unique participants (245 treatment, 229 control). Analysis of 13 primary outcomes (of 7 clinical trials) revealed a modest positive overall effect for symptom improvement or normalization of brain physiology (Hedges’ g = 0.308, 95% CI: 0.167, 0.448). Autism spectrum disorder studies showed the most consistent evidence (g = 0.264, 95% CI: 0.107, 0.421), while other conditions showed wider confidence intervals. Age-stratified analysis showed that adult populations (mean age 23.3 years, *n* = 5 outcomes) demonstrated higher effect sizes (g = 0.463, SD = 0.402) compared to pediatric populations (mean age 11.8 years, *n* = 8 outcomes; g = 0.318, SD = 0.212). Whole plant preparations (g = 0.328, 95% CI: 0.083, 0.573) and pharmaceutical cannabinoids (g = 0.292, 95% CI: 0.069, 0.515) showed comparable effects. CBD dosages ranged from 17.5 mg to 600 mg per day, with no significant correlation between dosage and effect size (ρ = -0.014, *p* = 0.963). Mild to moderate side effects were reported, but no serious adverse events. Risk of bias assessment ranged from low (*n* = 3) to high (*n* = 5).

**Conclusion:**

While meta-analysis of effect sizes for primary outcomes revealed modest positive effects, particularly for autism spectrum disorders, the current evidence remains insufficient to broadly recommend cannabinoids for treating mental disorders in youth populations. Larger, controlled studies with standardized outcomes are needed to establish definitive clinical recommendations.

**Supplementary Information:**

The online version contains supplementary material available at 10.1186/s13034-024-00846-5.

## Introduction

Interest in the therapeutic benefits of cannabis and specific cannabinoids has grown significantly since the 1990s, largely driven by an increased understanding of the endocannabinoid system (ECS) [1, 2]. In recent years there has been a global trend towards relaxing regulatory restrictions. Several countries and states have legalized recreational cannabis use, and many have approved its medicinal use [3] for adults. In some European countries, for instance in Switzerland, products containing cannabidiol (CBD) are available for purchase [4] for adult populations and regulated access to recreational cannabis is currently being investigated in several trials [5, 6].

In many countries, there has been a significant increase in recent years in medicinal cannabis (MC) prescriptions for mental health conditions [7, 8]. Notably, conditions such as autism spectrum disorder (ASD), anxiety disorder, and attention deficit hyperactivity disorder (ADHD) have seen an increased number of approvals despite a lack of definitive evidence supporting its efficacy [7]. Most of these psychiatric disorders emerge in childhood and early adolescence [9]. These trends underscore the rising interest in MC for pediatric neurodevelopmental and neuropsychiatric disorders, raising important questions about its efficacy and safety.

In child and adolescent psychiatry neurodevelopmental disorders (NDDs) are of interest due to their high prevalence [9]. NDDs are complex conditions that begin early in development and include ASD, ADHD, intellectual disability (ID) and motor disorders. These disorders exhibit wide genetic and clinical variability and are recognized as persistent, lifelong conditions that significantly impact quality of life and functioning into adulthood [10]. Co-occurring behavioral problems (BP), especially in children and adolescents with ID, present challenges for clinicians, caregivers and the socio-economic system [11, 12]. Despite the frequent prescription of psychotropic medications for BP, these treatments often show limited efficacy, poor adherence, and potential adverse effects in adults [13]. Efficacy and adverse effects of psychopharmacological treatment in children and adolescents are unfortunately still much less well understood [14, 15].

Research in child and adolescent psychopharmacology is gaining momentum, with newer modalities like ketamine, nitrous oxide, CBD, and other cannabis derivatives showing therapeutic potential. However, these treatments still lack comprehensive data on long-term safety and efficacy, limiting their current clinical use in pediatric populations [16]. Given the emerging interest in cannabinoids as potential therapeutic agents for psychiatric disorders in young people, further research is essential to determine their safety, efficacy, and appropriate dosing in children and adolescents [17].

In the past decades an increasing number of publications describing the use of cannabinoids to treat pediatric NDDs, neuropsychiatric and somatic conditions. However, synthesized evidence to assess efficacy and safety for the younger population is scarce [18]. In contrast, several reviews and meta-analyses have been conducted for adult populations [19–22]. Solmi et al. (2023) conducted an umbrella review investigating the risks and benefits of cannabis use [23]. They concluded that cannabis use is associated with poor mental health, cognitive impairment, increased risk of traffic accidents, and adverse effects during pregnancy, making it unsuitable for adolescents, young adults, pregnant women, and drivers. Conversely, CBD shows potential in reducing seizures in epilepsy [24] and cannabinoids may benefit chronic pain, multiple sclerosis, and cancer-related symptoms, though clinical guidelines require careful consideration of efficacy, safety, and patient information [25, 26]. Some surveys indicate high rates of cannabis use among individuals with conditions such as depression, anxiety and post-traumatic stress disorder, often for self-medication purposes [27, 28]. Adolescent use of commercial CBD for putative health benefits is also increasing [29]. While CBD seems safe for psychiatric symptoms, scholars agree that more research is needed before it can be recommended for psychiatric treatment [23]. Comprehensive reviews of RCTs focusing on the use of cannabinoids in children, adolescents and young adults are lacking.

This systematic review aims to address this gap by focusing on RCTs to evaluate the impact of cannabinoids on mental health outcomes, as well as their safety in children, adolescents, and young adults. By concentrating on RCTs, this review seeks to provide the highest level of evidence to guide future research and clinical practice regarding the use of cannabinoids in treating mental disorders in younger populations.

## Background

### Terminology in this review

In cannabinoid research and in the commercial cannabis industry, various forms of cannabis and its derivatives are used, each with distinct characteristics and applications [30, 31]. Commercially available cannabis products not regulated by medical quality control vary widely in cannabinoid content and ratios [32, 33]. Contrary, cannabis based medicinal products (CBMP), have undergone medical regulatory standards, such as for example Epidyolex^®^ (CBD) for severe treatment-resistant epilepsy [34]. Beyond tetrahydrocannabinol (THC) and CBD, the cannabis plant contains over 100 other cannabinoids and additional compounds like terpenes [35]. Alongside phytocannabinoids, there are also endogenous and synthetic cannabinoids [36]. This review analyzes RCTs involving whole-plant materials and pharmaceutically synthesized or extracted cannabinoids in different formulations. Whole-plant (WP) extracts typically specify a CBD: THC ratio, but also contain the full spectrum of cannabinoids and terpenes. In this review, analogue to Black et al. (2019), medicinal cannabis (MC) refers to any part of the cannabis plant, including buds, leaves, or complete plant extracts, used for therapeutic purposes [19]. This broad category encompasses a variety of plant-based preparations. In contrast, pharmaceutical cannabinoids (PC) include pharmaceutical-grade extracts with standardized levels of THC and CBD, as well as synthetic cannabinoid derivatives. Different CBD: THC ratios, such as for example 20:1 or 9:1, are used to balance therapeutic benefits and minimize THC’s psychoactive effects. For clarity in this review, the term cannabis treatment (CTx) is used to encompass both MC and PC. Abbreviations are also listed in Appendix I (See Appendix I).

### The endocannabinoid system (ECS)

The ECS is essential in the developing nervous system, facilitating communication between neurotransmitter systems and regulating various functions including psychiatric, neurological, cardiovascular, endocrine, and immune processes [37]. It also plays a role in cognition [38], mood [39, 40], and sleep [41] regulation. The ECS consists of G-protein-coupled receptors (cannabinoid receptor types 1 (CB1) and 2 (CB2)), lipid-based endocannabinoid neurotransmitters, and enzymes that synthesize and degrade these endocannabinoids [42]. CB1 receptors are prevalent in the central nervous system, but are also present in diverse organs including liver, adipose tissue and the skin. CB2 receptors are mainly found on immune cells within the central nervous, immune, and hematopoietic systems [42, 43]. Besides its role in response, synthesis and degradation of endocannabinoids, the receptors of the ECS are a primary target of THC [42]. Abnormal levels of endocannabinoids correlate with the severity of psychotic disorders [44] and play a key role in the pathophysiology of autism spectrum disorder (ASD) [45, 46], epilepsy [47] and multiple sclerosis [48].

### Tetrahydrocannabinol (THC) and cannabidiol (CBD)

THC is the best studied alkaloid of all cannabinoids. It is the primary psychoactive constituent in cannabis, that activates the CB1 receptor of the ECS [42]. THC disrupts the retrograde signaling systems of endocannabinoids and allosterically modulates opioid receptors. Acute administration of THC causes increased dopamine (DA) release and neuron activity, while long-term use is associated with blunting the DA system [49]. Following THC, CBD is the most extensively researched phytocannabinoid [50]. Although CBD engages with various receptor systems, the precise mechanisms underlying its proposed effects remain unclear. Unlike THC, CBD acts as an inverse agonist at the CB2 cannabinoid receptor and serves as a non-competitive modulator of the CB1 cannabinoid receptor. Unlike most antipsychotic drugs, CBD does not appear to exhibit DA receptor antagonistic properties [51–53]. CBD stimulates the serotonin 1 A receptor (5HT_1A_), inhibits adenosine reuptake, and enhances levels of the endocannabinoid anandamide [54]. The inhibition of glutamate release and partial agonism at the DA_2_ receptor are also considered critical in explaining its purported antipsychotic effects [55].

### Pharmacology, bodily impact and psychological effects

#### Pharmacokinetics of THC and CBD

Orally consumed THC is metabolized in the liver by CYP2C and CYP3A into the psychoactive 11-OH-THC and then into the inactive 11-COOH-THC [50]. About 65% of THC is excreted in feces and 20% in urine, with 80–90% eliminated within 5 days as hydroxylated and carboxylated metabolites [56]. THC’s bioavailability ranges between 4 and 12%. Due to its high lipid solubility, THC accumulates in fat tissues and is slowly released into circulation. The plasma half-life of THC is 1–3 days in occasional users and 5–13 days in chronic users [57]. CBD’s pharmacokinetics are complex, with major metabolites being hydroxylated 7-COOH derivatives excreted intact or as glucuronide conjugates [57]. The bioavailability of CBD varies by administration route: inhalation ranges from 11 to 45%, while oral bioavailability is about 6% [58, 59]. Like THC, CBD is highly lipid-soluble, rapidly distributing in the brain, adipose tissue, and other organs. Its half-life is estimated at 18–32 h [57]. CBD and THC influence drug metabolism by interacting with CYP P450 enzymes. THC induces CYP1A2 [60], while CBD inhibits CYP3A4 and CYP2D6 [61]. THC and CBD has been found in human breast milk, raising concerns about its impact on infant brain development [62, 63].

#### Pharmacodynamics, bodily impact and psychological effects of THC and CBD

Cannabis produces sedation and causes significant pharmacodynamic interactions when co-administered with other central nervous system (CNS) depressants [60]. Commonly, inhalation results in stronger psychoactive effects than ingestion, with higher THC concentrations in the brain compared to the blood [57]. Ethanol intoxication increases plasma THC levels and enhances subjective effects of smoking cannabis among healthy volunteers [61]. In healthy volunteers, THC induces psychotic symptoms [64], altered perception [65], increased anxiety [66], and cognitive deficits [67]. THC causes dose-dependent performance impairment, peaking within the first hour and decreasing over 2 to 4 h [68]. Cannabinoids may cause tachycardia with cardiac toxicity when combined with sympathomimetic agents [69]. CBD administration is associated with fatigue and somnolence, especially when combined with medications acting upon the CNS, and can mitigate THC-associated psychotropic and cardiovascular effects [70].

### Safety and toxicity

THC stimulates the mesolimbic DA system, potentially leading to addiction via reward pathway overactivation [71]. Recent molecular and neuroimaging studies reveal that adolescent cannabis exposure affects cortical development through alterations in synaptic and dendritic architecture, with significant impacts on gene expression patterns related to neuron projection development, learning, and memory processes [72]. Adolescent cannabis use heightens the risk of psychotic disorders, which seems dependent upon dose and age [73]. Conversely, some data suggest that CBD alleviate THC-induced psychotic symptoms [74, 75]. However, converse evidence found that CBD did not reduce THC’s effects challenge the idea that CBD can make cannabis “safer” [76]. Moreover, THC levels in cannabis correlate with the incidence of cannabis-induced psychotic disorders [77]. Aside from increased risk for a psychotic disorder, long term cannabis use has been associated with somatic conditions, such as diseases of the liver, lungs, heart and vasculature [78]. Furthermore it may result in cognitive deficits [79, 80], anxiety [81], and development of cannabis use disorder [82]. Despite its proclaimed putative therapeutic potential, CBD is certainly not without risks [75]. Animal studies have shown adverse effects such as developmental toxicity, CNS inhibition and hepatocellular injury, typically at doses higher than those used in human therapy [83]. Human studies have reported drug interactions, hepatic abnormalities, diarrhea, fatigue, vomiting, and somnolence [84].

## Methods

This systematic review is reported according to the Preferred Reporting Items for Systematic Reviews and Meta-Analysis (PRISMA) guidelines [85, 86]. The protocol has been pre-registered on INPLASY (INPLASY202330017) https://inplasy.com/inplasy-2023-3-0017/ (doi: 10.37766/inplasy2023.3.0017).

### Data acquisition

The data for this systematic review were gathered according to the PRISMA guidelines for Systematic Reviews and Meta-Analyses. The main aim was the identification of all human randomized controlled trials (RCT) where cannabinoids have been used with therapeutic intention within the medical field of child, adolescent, and transitional age psychiatry until a maximum mean age of 25 years. Only studies published until 30th of June 2024 were considered.

### Search strategy

For data acquisition we performed systematic electronic searches using PubMed, Europe PubMed Central and EMBASE. We searched for a combination of the following search terms in each of the three databases: (‘child’ OR ‘adolescent’ OR ‘youth’) AND (‘cannabis’ OR ‘cannabidiol’ OR ‘cannabinoid’) AND (‘psychiatry OR ‘treatment’ OR ‘therapy’). Furthermore, we applied this combination of keywords: (‘child’ OR ‘child’/exp OR child) AND (‘adolescent psychiatry’/exp OR ‘adolescent psychiatry’ OR ((‘adolescent’ OR ‘adolescent’/exp OR adolescent) AND (‘psychiatry’ OR ‘psychiatry’/exp OR psychiatry))) AND (‘cannabinoid’ OR ‘cannabinoid’/exp OR cannabinoid) AND (‘treatment’ OR ‘treatment’/exp OR treatment).

### Eligibility criteria

We determined eligibility criteria in accordance with the following PICOS:

#### Population

Children, adolescents, or young adults until a maximum mean age of 25 years of the cannabinoid treatment group. Any neurodevelopmental and/or psychiatric disorder including multiple co-occurring psychiatric disorders were eligible for inclusion provided clear diagnostic criteria were reported. Epilepsy or other disorders outside the psychiatric context were excluded. Somatic comorbidities were considered if (a) cannabinoid treatment was primarily applied for psychiatric symptoms and (b) medical conditions did not significantly interfere with the interpretation of psychiatric outcomes. Studies were required to report participants’ relevant medical conditions and concurrent medications.

#### Intervention/exposure

Only published RCTs with therapeutic application of cannabinoids within the field of psychiatry and focusing on children, adolescents, or young adults were considered.

#### Comparisons

The main comparators were pre-treatment and post-treatment symptom changes and/or normalization of brain physiology.

#### Outcomes

Pre- and post-intervention measures and effect sizes were considered as primary outcomes. Secondary outcomes were dosages and adverse events.

### Data extraction and risk of bias assessment

**Fig. 1:**
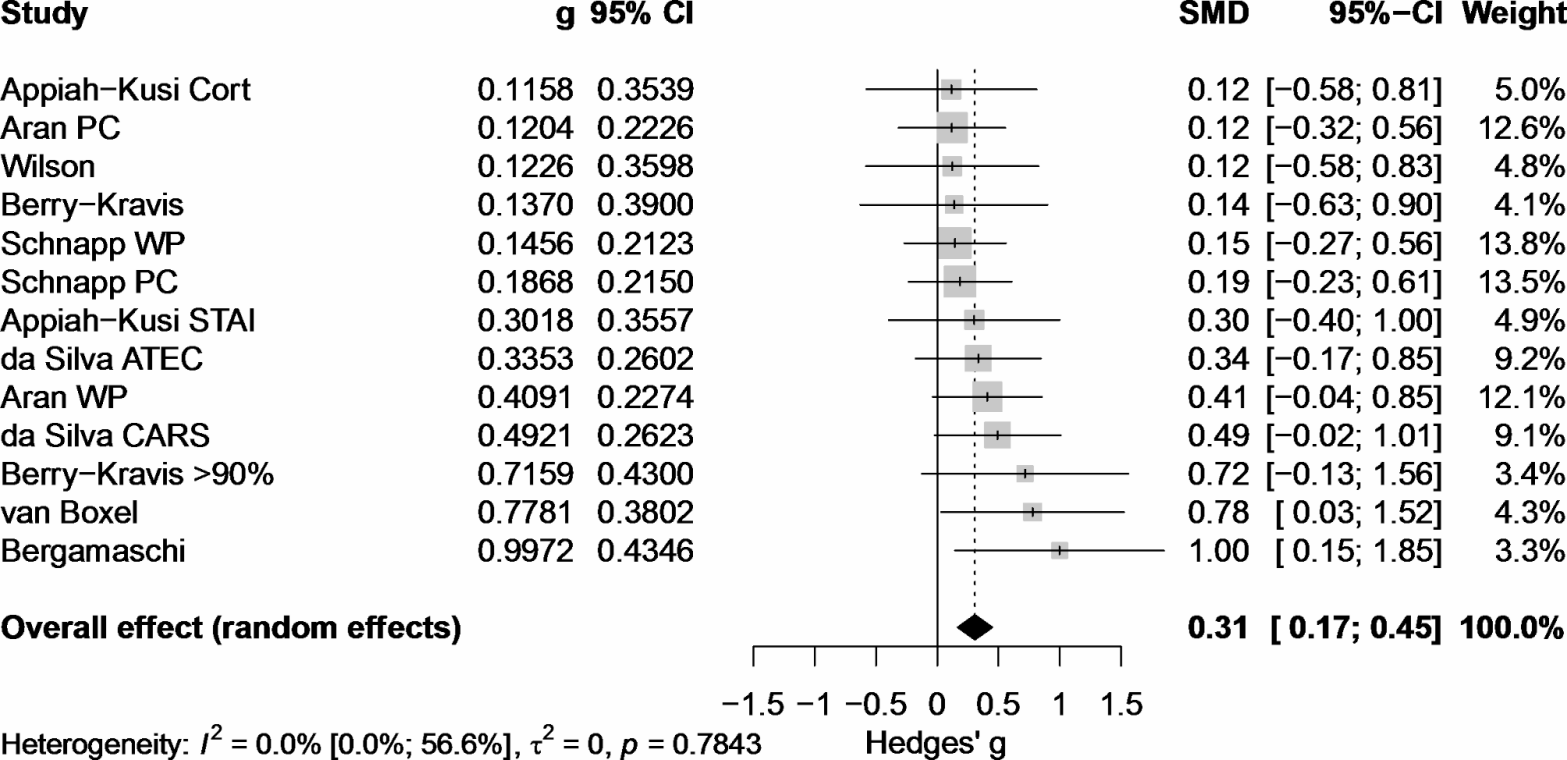
Forest plot for Hedges’ g. Forest plot showing standardized mean differences (Hedges’ g) with 95% confidence intervals for cannabinoid treatment versus placebo across studies. The size of squares represents the relative weight of each study in the meta-analysis based on inverse variance weighting. Horizontal lines indicate 95% confidence intervals. The black diamond at the bottom represents the overall effect (random effects] Hedges’s g = 0.308 (0.167;0.448). Values to the right of the zero line indicate favorable effects of cannabinoid treatment. WP = Whole Plant extract, PC = Pure Cannabinoid, ATEC = Autism Treatment Evaluation Checklist, CARS = Childhood Autism Rating Scale, Cort = Cortisol, STAI = State-Trait Anxiety Inventory, DMN = Default Mode Network, VAMS = Visual Analogue Mood Scale. *Berry-Kravis subgroup analysis includes participants with > 90% methylation

**Fig. 2:**
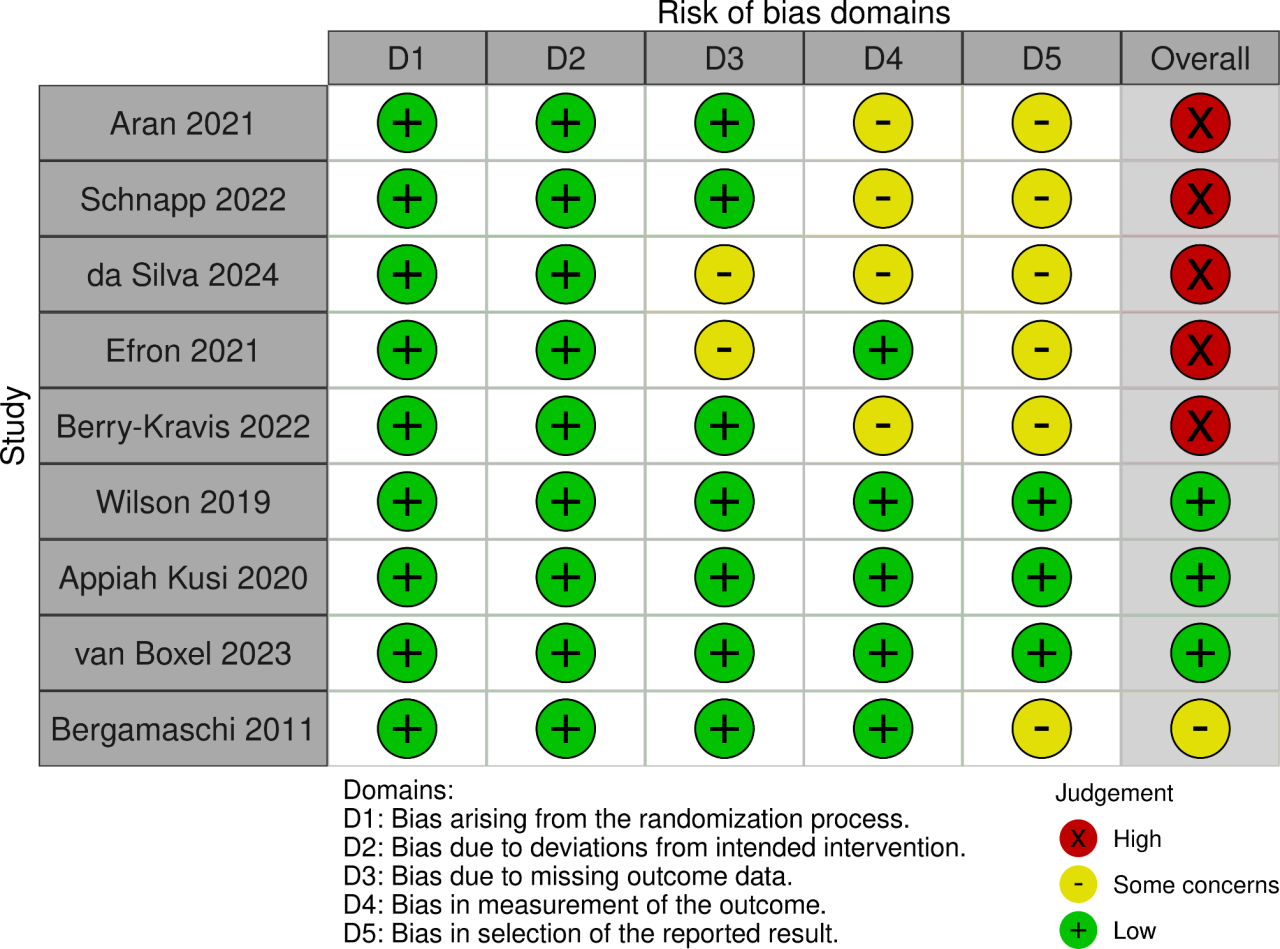
Cochrane Risk of Bias 2 tool (RoB2) for Cannabinoids for psychiatric symptoms in youth

All electronically gathered data were screened and assessed by two independent reviewers (rater 1 AB, rater 2 PK). For data collection, screening, and assessment we used nested knowledge (www.nested-knowledge.com). The platform nested knowledge was used to organize the literature database and keep track of the screening process. Abstract screening was performed by rater 1 and 2. Consequently, full text screening and inclusion decisions were performed by rater 1 and 2. From the articles included we recorded author names and date, disorder type, number of participants (total, healthy controls (HC), CTx, placebo (PLB)), age range, mean age (Table 1); specification on cannabis applied within the RCT, formulation, application route (Table 2); study duration, objectives/aims, exclusion criteria, questionnaires/comparators used within the trial (Table 3), statistical significances, results (Table 4), information on cannabis naivety, concomitant medications and adverse events (Table 5), and effect sizes (Table 6; Fig. 1). For the analysis of risk of bias for all studies we used the Cochrane Risk of Bias 2 (RoB 2)-tool and assessment guidelines [87]. Figure 2 was created with *robvis* [88].

### Meta-analysis for effect sizes of primary outcomes

In addition to the qualitative systematic review a meta-analysis for effect sizes of primary outcomes was conducted. Our meta-analysis was conducted using R (Version 4.2.3) and RStudio (2023.09.1 + 494) [88], primarily utilizing the ‘meta’ (Version 7.0–0) and ‘metafor’ (Version 4.2-0) packages. Effect sizes were calculated as Hedges’ g to correct for small sample size bias in Cohen’s d. For studies reporting change scores, post-only comparisons, least square means (LSM), pre-post measurements, and F-statistics, appropriate effect size calculations were implemented using custom functions in R. The random-effects model was fitted using restricted maximum likelihood estimation (REML) with Knapp-Hartung adjustments. Heterogeneity was assessed using Q-statistics, I², and τ².

Subgroup analyses were performed for treatment type (WP vs. PC), study duration (short-term vs. long-term), and clinical indication (ASD, BP, CHR, and anxiety). Additional sensitivity analyses were conducted using different correlation coefficients (*r* = 0.3 and *r* = 0.7) for the calculation of pre-post effect sizes.

For the analysis of dosage-effect and age-effect relationships, we employed additional R packages including ‘correlation’ for Spearman rank correlations, ‘ggplot2’ (Version 3.4.3) for visualization of relationships between variables, and ‘dplyr’ (Version 1.1.3) and ‘tidyr’ (Version 1.3.0) for data transformation and summary statistics. Age-stratified analyses were conducted to examine effect size variations between pediatric and adult populations, while controlling for potential confounding factors such as dosage and formulation type. Correlational analyses were performed to assess the relationships between CBD dosage, age, and treatment effects across different formulation types and clinical indications.

## Results

### Selection of records

**Fig. 3:**
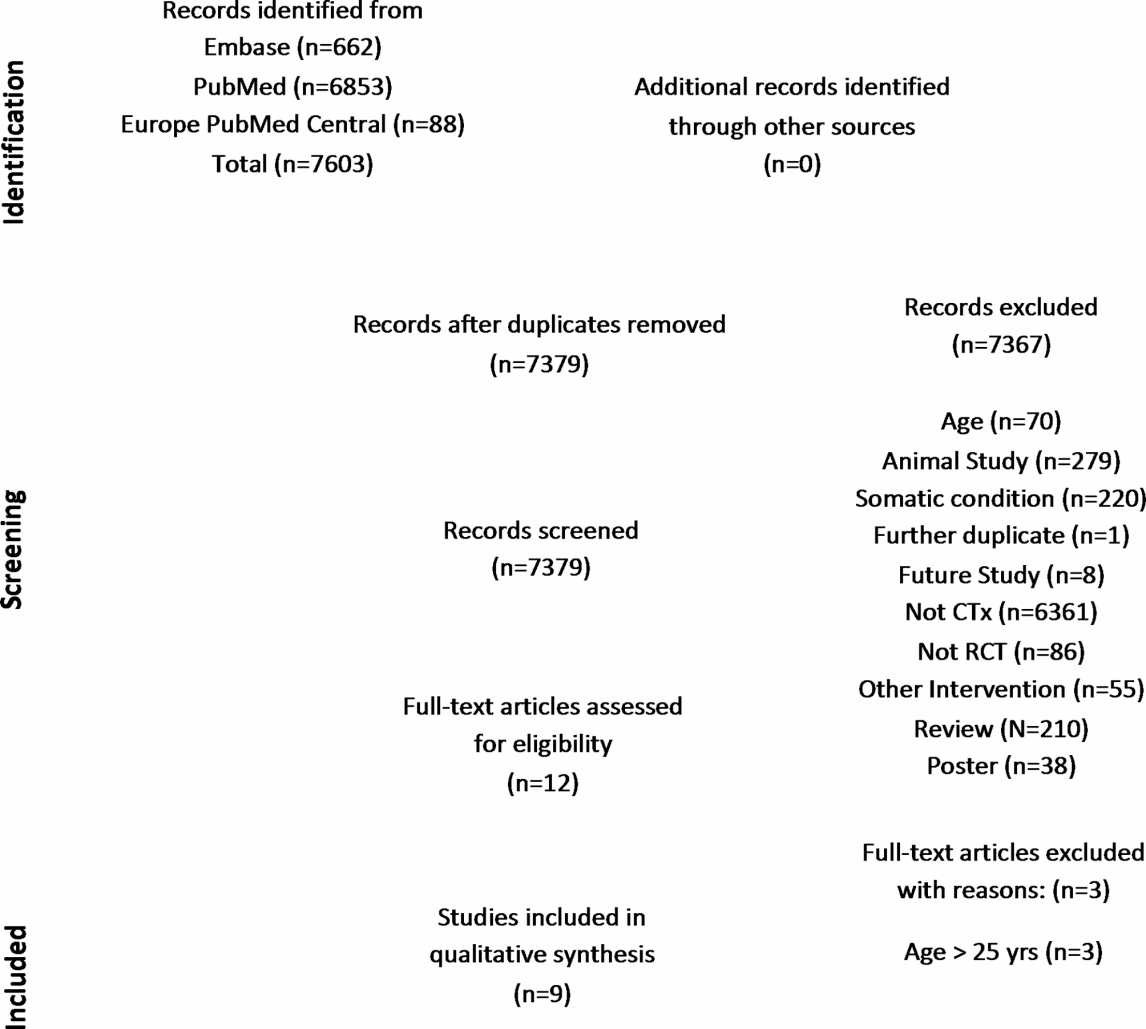
Flow diagram according to PRISMA 2020

Following the PRISMA guidelines [85, 86] we performed data acquisition and analysis for inclusion and exclusion of relevant literature. As per 30th of June 2024, we identified 7603 records through performance of database searches (PubMed, Embase). After deduplication, 7379 records were screened, of which nine publications of eight RCTs met the inclusion criteria (see flowchart in Fig. 3). We provided the main information of the identified studies in Tables 1, 2, 3, 4, 5 and 6. See Appendix I for abbreviations used. Please see supplementary files for the complete RStudio code and raw data.

### Randomized placebo-controlled studies

In total we identified nine RCTs which were included in this review, however two publications referred to a single clinical trial [89, 90]. Thus, eight RCTs were analyzed; all of them were double-blinded. In our Tables (1, 2, 3, 4, 5 and 6) we clustered the studies into “disorder types”: (i) Three studies of cannabinoid treatment (CTx) for symptoms of autism spectrum disorders (ASD) (ii) two studies of CTx for behavioral problems (BP) (iii) three studies focusing on CTx for psychotic disorders and/or clinical-high-risk for psychosis (PSY) and (iv) one study of CTx for anxiety (ANX). Results were qualitatively synthesized, and quantitative analysis was conducted for primary outcomes where possible.

**Table 1:** Study overview: participants receiving cannabinoids vs. placebo, age range

	Author	Disorder	Number of particpants (*N*)				Age range	Mean age
			Total	HC	CTx	PLB		
1	Aran et al. (2021) [89]*	ASD	150	0	100	50	5–21	11.8
2	Schnapp et al. (2022) [90]*	ASD	150	0	100	50	5–21	11.8
3	da Silva Junior et al.(2024) [91]	ASD	60	0	31	29	5–11	7.68
4	Efron et al. (2020) [92]	BP	8	0	4	4	8–16	13.9
5	Berry-Kravis et al. (2022) [93]	BP	212	0	110	102	5–21	9.70
6	Wilson et al. (2019) [95]	PSY	52	19	16	15	18–35	22.7 **
7	Appiah-Kusi et al. (2020) [94]	PSY	58	26	16	16	Not stated	22.33 **
8	Van Boxel et al. (2023) [96]	PSY	31	0	16	15	Not stated	24.7 **
9	Bergamaschi et al. (2011) [97]	ANX	36	12	12	12	Not stated	24.6 **

Dx = diagnosis/disorder type, HC = Healthy control, CTx = Cannabinoid Treatment, PLB = Placebo, MC = Medicinal cannabis, PC = Pharmaceutical cannabis, CBD = Cannabidiol, THC = Tetrahydrocannabinol; ASD = Autism Spectrum Disorder, BP = Behavioral problems, PSY = Psychotic disorders; being at clinical high risk for psychosis, ANX = Anxiety disorder; * Aran et al. (2021) and Schnapp et al. (2022) evaluated the same participants but different research questions, ** mean ages of the CTx-groups of respective studies

**Table 2:** Study overview: cannabinoid type, dosage, formulation and application

	Author	Dx	CTx (MC/ PC)	CBD	THC	CBD: THC	Max. CBD/d in mg	Max. THC/d in mg	Form	Application
1	Aran et al. (2021) [89]*	ASD	MC and PC	Yes	Yes	20:1	420	21	Oil	Oral
2	Schnapp et al. (2022) [90]*	ASD	MC and PC	Yes	Yes	20:1	420	21	Oil	Oral
3	da Silva Junior et al.(2024) [91]	ASD	MC	Yes	Yes	9:1	17.5	1.95	Oil	Oral
4	Efron et al. (2020) [92]	BP	PC	Yes	No	n.a.	1000	n.a.	Oil	Oral
5	Berry-Kravis et al. (2022) [93]	BP	PC	Yes	No	n.a.	500	n.a.	Gel	Topical
6	Wilson et al. (2019) [95]	PSY	PC	Yes	No	n.a.	600	n.a.	Caps.	Oral
7	Appiah-Kusi et al. (2020) [94]	PSY	PC	Yes	No	n.a.	600	n.a.	Caps.	Oral
8	Van Boxel et al. (2023) [96]	PSY	PC	Yes	No	n.a.	600	n.a.	Caps.	Oral
9	Bergamaschi et al. (2011) [97]	ANX	PC	Yes	No	n.a.	600	n.a.	Caps.	Oral

Dx = Diagnosis/disorder type; HC = Healthy control, CTx = Cannabinoid Treatment, PLB = Placebo, MC = Medicinal cannabis, PC = Pharmaceutical cannabis, CBD = Cannabidiol, THC = Tetrahydrocannabinol; Max. = Maximum, form = Formulation, caps. = Capsule, Application = Application route, mg = milligram; ASD = Autism Spectrum Disorder, BP = Behavioral problems, PSY = Psychotic disorders; being at clinical high risk for psychosis, ANX = Anxiety disorder; * Aran et al. (2021) and Schnapp et al. (2022) evaluated the same participants but different research questions

### Cannabinoid treatment for autism spectrum disorder (ASD)

A study by Aran et al. (2021) investigated the effects of CTx on ASD in a randomized trial, with a focus on the Clinical Global Impression - Improvement (CGI-I) scale. The trial included 150 participants (mean age = 11.8, age range 5–21) diagnosed with ASD according to DSM-5 criteria and moderate to severe BP. The participants were divided into three groups: one receiving whole-plant cannabis extract (MC, *n* = 50), another receiving purified cannabinoids (PC, *n* = 50), and a PLB group (*n* = 50). Primary and secondary outcomes were changes in total scores of the home situations questionnaire (HSQ-ASD) and Autism Parenting Stress Index (APSI) which did not differ among groups. However, their analysis showed that disruptive behavior significantly improved in 49% of the whole-plant extract group compared to 21% in the PLB group (*p* = 0.005), as measured by the clinician-rated CGI-I scale. In contrast, the PC-group did not show a statistically significant improvement compared to the PLB-group (*p* = 0.08). The study concluded that while the treatment was well-tolerated, the evidence for its efficacy was mixed and insufficient [89].

Schnapp et al. (2022) studied the same population as part of the previously described trial by Aran et al. (2021). Their research question focused on the effects CTx on sleep disturbances in children and adolescents ASD as compared to PLB. The trial included 150 participants (mean age = 11.8, age range 5–21) diagnosed with ASD confirmed by Autism Diagnostic Observation Schedule (ADOS-2), and who had moderate to severe BP. Participants were divided into three groups: MC (*n* = 50), PC (*n* = 50) and PLB (*n* = 50). To evaluate sleep scores a total of *n* = 131 participants completed pre- and post-treatment measurements for twelve weeks. The results showed that CTx was not superior to PLB in improving sleep parameters measured by the Children’s Sleep-Habit Questionnaire (CSHQ), including bedtime resistance, sleep-onset delay, and sleep duration. However, improvements in the CSHQ total score, regardless of treatment type, were associated with improvements in core autistic symptoms, as indicated by the Social Responsiveness Scale (SRS) total scores (Period 1: *r* = 0.266, *p* = 0.008; Period 2: *r* = 0.309, *p* = 0.004). The study concluded that while sleep improvements were not higher with CTx compared to PLB, further research is needed. Additionally, the study found no significant differences in the frequency of mild to moderate adverse events between the CTx and PLB groups, and no treatment-related severe or serious adverse events were reported [90].

The most recent RCT was a study by da Silva Junior et al. (2024) which evaluated the efficacy and safety of a CBD-rich cannabis extract (MC) in children with ASD. Over 12 weeks 60 study participants (mean age = 7.68, age range 5–11) received either CTx (*n* = 31) or PLB (*n* = 29). Their results showed significant improvements in social interaction (*p* = 0.0002), anxiety (*p* = 0.016), psychomotor agitation (*p* = 0.003), and meal frequency (*p* = 0.04). Mild adverse effects, such as dizziness, insomnia, colic, and weight gain, were reported in 9.7% of the participants, with no serious adverse events. The findings suggest that CTx extract may offer therapeutic benefits for ASD symptoms, warranting further research to confirm these results [91].

### Cannabinoid treatment for behavioral problems (BP)

A small pilot-study by Efron et al. (2020) aimed to investigate the feasibility of conducting a RCT to assess the efficacy of pharmaceutical CBD in reducing severe BP in children and adolescents with intellectual disability (ID). The study enrolled eight participants (mean age = 13.9, age range 8–16), who were randomly assigned to receive either 98% CBD in oil (*n* = 4) or PLB (*n* = 4) for 8 weeks. The CBD dosage was gradually increased over nine days to a maximum of 20 mg/kg bodyweight per day, divided into two daily doses, with a maximum of 1000 mg per day. All participants (*N* = 8) completed the full study protocol with no serious adverse events or drop-outs. Adherence to the study protocol was good (100% adherence to study visits and medication, 92% completion of blood tests, 88% completion of questionnaires). Parents reported high acceptability of the study design, and all indicated they would recommend the treatment to other families. The study observed an efficacy signal in favor of the CTx, with improvements in all assessed questionnaires being more pronounced in the CBD-group compared to PLB. These findings suggest that the trial protocol is feasible and well-accepted, and there is potential therapeutic efficacy of CBD in managing severe BP in children with ID. However, due to the very small sample size, further research with is needed [92].

Another RCT by Berry-Kravis et al. (2022) evaluated the efficacy and safety of ZYN002 CBD transdermal gel in children and adolescents with Fragile X Syndrome (FXS). The study involved 212 participants (mean age = 9.7; age range 5–21), who were diagnosed with FXS based on molecular documentation of the full FMR1 mutation. Participants were required to meet specific criteria related to BP and were excluded if they used cannabis products or certain medications. The trial administered ZYN002 in doses of 250 mg/day or 500 mg/day for 12 weeks (*n* = 110), depending on body weight or PLB (*n* = 102). While the primary endpoint (changes in social avoidance) was not achieved in the full cohort, significant improvements were observed in patients with ≥ 90% methylation of the FMR1 gene (*n* = 169; 79.7% of study population. ZYN002 was well-tolerated with a favorable benefit-risk profile, and about half of the participants experienced mild to moderate treatment-emergent adverse events, primarily application site pain, with no serious adverse events reported [93].

### Cannabinoid treatment for psychotic disorders or clinical-high-risk states (CHR)

Appiah-Kusi et al. (2020) investigated the effects of short-term CBD treatment on stress response in individuals at clinical high risk (CHR) of developing psychosis. The study included 58 participants divided into three groups: CHR patients on CBD (*n* = 16; mean age = 22.3), CHR patients on PLB (*n* = 17, mean age = 25.12), and HC (*n* = 25, mean age 23.91). Over seven days, CHR patients received capsules of 600 mg of CBD (PC). On the eight day a Trier Social Stress Test (TSST) was performed. Intermediate cortisol reactivity and anxiety levels were measured via State Trait Anxiety Inventory (STAI) and Self-Statements during Public Speaking Scale (SSDPS). Results were compared to HC and PLB-treated CHR patients during stress exposure. The study found that cortisol reactivity to stress was highest in HC, lowest in CHR patients on PLB, and intermediate in CHR patients treated with CBD. Significant differences were observed between the HC and both CHR groups, but not between the CHR groups themselves. CBD-treated CHR patients also showed intermediate anxiety and stress levels compared to the other groups. The findings suggest that CBD may help mitigate stress responses, especially cortisol reactivity, in CHR individuals, highlighting the need for further research with larger sample sizes and longer trial duration [94].

Another study by Wilson et al. (2019) explored the effects of a single 600 mg dose of CBD on insular dysfunction during motivational salience processing in CHR. The study involved 52 participants, comparing fMRI responses during a monetary incentive delay task between antipsychotic-naive CHR patients and HC (*n* = 19, mean age = 23.9). Results indicated that CHR patients on PLB (*n* = 15, mean age = 24.1) exhibited increased activation in the insular cortex as compared to HC, while CBD treatment of CHR patients (*n* = 16, mean age = 22.7) appeared to attenuate this activation, suggesting a potential moderating effect of CBD on abnormal brain activity associated with psychosis risk. CBD reduced the heightened activation in the left insula/parietal operculum and was linked to a general slowing of reaction time. This, according to the authors, indicates a potential mechanism for its proposed antipsychotic effects by normalizing motivational salience and regulating motor responses [95].

The most recent study investigating CTx for psychotic disorders van Boxel et al. (2023) examined the effects of CBD on patients with recent-onset schizophrenia or related psychotic disorders. The study involved 31 patients who were treated with either 600 mg of CBD (*n* = 16; mean age = 24.7) or a PLB (*n* = 15, mean age = 27.5) daily for 28 days, alongside a stable dose of one antipsychotic agent. The research focused on changes in resting-state functional connectivity, prefrontal metabolite levels, and reward processing. Results showed that CBD increased connectivity in the default mode network (DMN) compared to PLB but did not significantly affect prefrontal metabolite concentrations or reward processing brain activity. However, exploratory analyses indicated that CBD might reduce positive symptom severity by lowering prefrontal glutamate and N-acetyl-aspartate (NAA) levels. Specifically, the Positive and Negative Syndrome Scale (PANSS) scores for positive symptoms correlated with decreases in glutamate and NAA levels in the CBD group but not in the PLB group [96].

### Cannabinoid treatment for anxiety disorders

Bergamaschi et al. (2011) investigated the effects of oral CBD in a short public speaking sequence in patients with Social Anxiety Disorder (SAD). A total of 24 treatment-naive young adults with SAD were randomly allocated to receive either 600 mg of CBD (*n* = 12, mean age = 24.6) or a PLB (*n* = 12, mean age = 22.9) in a double-blind design 1.5 h before the test. Additionally, HC (*n* = 12, mean age = 23.3) performed the speaking sequence without receiving any medication. Each participant took part in only one experimental session. Subjective ratings were measured using the Visual Analogue Mood Scale (VAMS) and the Negative Self-Statement Scale (SSPS-N), alongside physiological measures such as blood pressure, heart rate, and skin conductance, at six different time points during the speaking sequence. Their study found that pretreatment with CBD significantly reduced anxiety, cognitive impairment, and discomfort during speech performance, and significantly decreased alertness during anticipatory speech. In contrast, the PLB group exhibited higher levels of anxiety, cognitive impairment, discomfort, and alertness compared to the control group, as assessed with the VAMS. The SSPS-N scores increased significantly during testing in the PLB group, but this increase was almost abolished in the CBD group. No significant differences were observed between the CBD group and HC in SSPS-N scores or in the cognitive impairment, discomfort, and alert factors of VAMS. Overall, the increase in anxiety induced by the speaking sequence in subjects with SAD was reduced with the use of CBD, resulting in responses similar to those of HC [97]. See Tables 3 and 4 for study objectives and main results.

**Table 3:** Study overview: description of objectives, exclusion criteria, questionnaires

	Author	Dx	Duration	Objectives/ aims	Exclusion criteria	Questionnaires, Comparators
1	Aran et al. (2021) [89]*	ASD	12 w	Assess the superiority of CTx over PLB in ASD associated BP Comparison between MC/PC and PLB as a secondary outcome	Psychotic disorders Former CTx Medical condition Changes Tx 4 weeks prior	ADOS-2, VABS, CARS, HSQ-AS, SRS, CGI-I
2	Schnapp et al. (2022) [90]*	ASD	12 w	Impact of a CBD-rich CTx on sleep in patients with ASD	Psychotic disorders Current or former CTx	ADOS-2, CARS, SRS, CSHQ scores, VABS, CGI-I
3	da Silva Junior et al.(2024) [91]	ASD	12 w	Evaluation of the efficacy and safety of MC in ASD	Somatic comorbidities Cannabis prior to trial	ATEC, CARS, semi-structured interview
4	Efron et al. (2020) [92]	BP	8 w	Feasibility of a RPCT to reduce SBP in children with ID	Psychiatric comorbidities Anti-epileptic med. interacting with CBD Cannabis prior to trial	ABC-1; CASP; SDSC; FQOL; AQoL; DASS; APSI; CHU9D, SCQ, A-TAC, WASI-II, VABS, MOSES
5	Berry-Kravis et al. (2022) [93]	BP	12 w	Efficacy and safety of transdermal CBD gel in Fragile-X-Syndrom and BP	Liver/renal problems Positive drug screen Certain antiepileptic drugs / use of a strong CYP P450 3A4 inhibitor/inducer	ABC-C FXS, CaGI-S, CaGI-C
6	Wilson et al. (2019) [95]	PSY	1 d	Comparing salience network activation in the ACC and insular cortex in CHR vs. HC after single dose of CBD	Psychosis/mania Substance dependence (except cannabis), Neurological disorder or severe intercurrent illness	CAARMS; fMRI, reaction time, reward, delayed response
7	Appiah-Kusi et al. (2020) [94]	PSY	7 d	Assessment of short-term CTx for normalisation of acute neuroendocrine and anxiety response in CHR patients	Psychiatric disorder	STAI, SSDPS, cortisol reactivity in the TSST
8	Van Boxel et al. (2023) [96]	PSY	28 d	Impact of adjunctive CTx on functional connectivity, metabolite levels, reward processing in recent-onset psychotic disorder	Unstable antipsychotic Tx Corticosteroids, NSAI, drugs interacting with CBD Somatic and neurological disorders, IQ < 70 Substance use	PANSS, HAM-D, YRMS, CGI, GAF, SOFAS, BACS, WHO-Assist, MAQ, fMRI
9	Bergamaschi et al. (2011) [97]	ANX	1 d	Effects of simulation public speaking test in HC and SAD patients receiving a single dose of CBD vs. PLB	Non treatment-naive Other concomitant psychiatric disorder Neurological disorders Substance abuse	VAMS, SSPS, BSS; SPIN; skin conductance, blood pressure, heart rate

Dx = Diagnosis/disorder type; CTx = Cannabinoid Treatment, PLB = Placebo, HC = Healthy control, SBP = Severe behavioral problems, ID = Intellectual disability, CHR = Clinical high risk for psychosis, ASD = Autism spectrum disorder, SAD = Social anxiety disorder, CBD = Cannabidiol, THC = Tetrahydrocannabinol, MC = medicinal cannabis, PC = pharmaceutical cannabis, ACC = Anterior cingulate cortex, PANSS = Positive and Negative Syndrome Scale, HAM-D = Hamilton Depression Scale, YMRS = Young Mania Rating Scale, CGI = Clinical Global Impression, GAF = Global Assessment of Functioning scale, SOFAS = Social and Occupational Functioning Assessment Scale, BACS = Brief Assessment of Cognition in Schizophrenia, WHO Assist 3.0 = World Health Organization Alcohol, Smoking, and Substance Involvement Screening Test, MAQ = Medication Adherence Questionnaire, CARS = Childhood Autism Rating Scale, ABC = Aberrant Behaviour Checklist, SCQ = Social Communication Questionnaire, A-TAC = Autism-Tics ADHD and Comorbidities Inventory, MOSES = Monitoring of Side Effects Scale, CHU-9D = Child Health Utility-9D, WASI-II = Wechsler Abbreviated Scale of Intelligence-II, Vineland-3, SDSC = Sleep Disturbance Scale for Children, AQoL = Assessment of Quality of Life, FQoL = Family quality of Life, DASS = Depression Anxiety Stress Scale, APSI = Autism Parenting Stress Index, CASP = Child and Adolescent Scale of Participation, CSHQ = Children’s Sleep-Habit Questionnaire, CGI-I = Clinical Global Impression-Improvement scale, SRS = Social Responsiveness Scale, ADOS-2 = Autism Diagnostic Observation Schedule, VABS = Vineland Adaptive Behavior Scales, HSQ-AS = Home Situations Questionnaire-ASD, ABC-C FXS = Aberrant Behavior Checklist-Community Edition FXS, CaGI-S = Caregiver Global Impression-Severity, CaGI-C = Caregiver Global Impression-Change, HSQ-ASD = Home Situations Questionnaire-Autism Spectrum Disorder, VAMS = Visual Analogue Mood Scale, SSPS = Self-Statements during Public Speaking Scale, BSS = Bodily Symptoms Scale, SPIN = Social Phobia Inventory, CAARMS = Comprehensive Assessment of At-Risk Mental States, fMRI = functional magnetic resonance imaging, DMN = Default mode network, ATEC = Autism Treatment Evaluation Checklist. * Aran et al. (2021) and Schnapp et al. (2022) evaluated the same participants but different research questions

**Table 4:** Description of statistical findings and results

	Author	Dx	Statistical significances	Results
1	Aran et al. (2021) [89]*	ASD	No significant changes in HSQ-ASD (primary-outcome) and APSI (secondary-outcome). Disruptive behavior on the CGI-I (co-primary outcome) was much/very much improved in 49% on MC (*n* = 45) versus 21% on PLB (*n* = 47; *p* = 0.005). Median SRS Total Score (secondary outcome) improved by 14.9 on MC (*n* = 34) versus 3.6 points after PLB (*n* = 36); *p* = 0.009.	Disruptive behavior improved significantly with MC vs. PLB SRS total score improved more with MC vs. placbeo No significant differences in total scores of HSQ-ASD and APSI
2	Schnapp et al. (2022) [90]*	ASD	CTx AND PLB improved the CSHQ total score and was associated with improvements in the autistic core symptoms, indicated by SRS total scores (Period 1: *p* = 0.008; Period 2: *p* = 0.004)	CTx did not show superiority over PLB in sleep Improvements in sleep were linked to bettered autistic core symtpoms
3	da Silva Junior et al.(2024) [91]	ASD	Significant results were found for social interaction ( *p* = 0.0002), anxiety ( *p* = 0.016), psychomotor agitation (F1,116 = 9.22, *p* = 0.003), number of meals a day (*p* = 0.04), and concentration (*p* = 0.01)	CBD-rich cannabis extract improved social interaction, anxiety and psychomotor agitation
4	Efron et al. (2020) [92]	BP	Not possible due to low number of included participants	Feasibile and accepted by participants and care-givers Efficacy signal in favor of active drug Caregivers showed high interest in participation
5	Berry-Kravis et al. (2022) [93]	BP	No statistical signifcance for primary endpoint for full cohort Signifcant improvement only in ≥ 90% methylation of FMR1 ( *p* = 0.020), in CaGI-C in SA and isolation, irritable and disruptive behaviors, and social interactions (p-values: *P* = 0.038, *P* = 0.028, and *P* = 0.002)	CTx showed significant improvement in social avoidance in patients with > 90% methylation Patients with 90% methylation showed superior results
6	Wilson et al. (2019) [95]	PSY	CAARMS subscale comparisions revealed no significant differences. Analysis of fMRI data showed significant activation differences in insula and frontal operculum regions Positive correlation between CAARMS score and left insula activation.	No significant differences in symptom subscales observed CBD attenuated insular activation, correlated with psychotic symptoms and salience perception CHR showed abnormal insular activation vs. HC
7	Appiah-Kusi et al. (2020) [94]	PSY	Cortisol reactivity group effect HC vs. CHR-PLB vs. CHR-CBD (*p* = 0.005) and linear decrease (*p* = 0.003). Across groups changes in anxiety and experience of public speaking stress (all p’s < 0.02) were greatest in the CHR-P and least in the HC, with CHR-CBD participants demonstrating an intermediate level of change	CHR-CBD showed intermediate cortisol response compared to HC and CHR-PLB CHR particpants had higher cannabis use than HC CBD may affect neuroendocrine response of acute stress
8	Van Boxel et al. (2023) [96]	PSY	CTx significantly changed functional connectivity in the DMN (DMN; time × treatment interaction *p* = 0.037), with increased connectivity in the CBD-group and reduced connectivity in the placebo group. Decreased positive symptom severity associated with diminishing glutamate (*p* = 0.029) and N-acetyl-aspartate (NAA; neuronal integrity marker) levels (*p* = 0.019) in the CBD, but not PLB group	CBD altered DMN connectivity in recent-onset psychosis patients No significant impact on prefrontal metabolite leves or reward processing No effect upon brain activity during reward processing
9	Bergamaschi et al. (2011) [97]	ANX	Significant differences among the groups were found in the mean scores of SPIN ( *p* = 0.001). The SPIN scores were significantly lower in healthy volunteers than in subjects with SAD who received CBD or PLB. No other significant differences were observed between the two groups with SAD	Reduced anxiety, cognitive impairments and discomfort in SAD patients Decreased alert levels in anticipatory speech for SAD patients Inhibited fear of public speaking in SAD patients Improved self-evaluation during public speaking in SAD patients

Dx = Diagnosis/disorder type; CTx = Cannabinoid Treatment, PLB = Placebo, HC = Healthy control, SBP = Severe behavioral problems, ID = Intellectual disability, CHR = Clinical high risk for psychosis, ASD = Autism spectrum disorder, SAD = Social anxiety disorder, CBD = Cannabidiol, THC = Tetrahydrocannabinol, MC = medicinal cannabis, PC = pharmaceutical cannabis, ACC = Anterior cingulate cortex, PANSS = Positive and Negative Syndrome Scale, HAM-D = Hamilton Depression Scale, YMRS = Young Mania Rating Scale, CGI = Clinical Global Impression, GAF = Global Assessment of Functioning scale, SOFAS = Social and Occupational Functioning Assessment Scale, BACS = Brief Assessment of Cognition in Schizophrenia, WHO Assist 3.0 = World Health Organization Alcohol, Smoking, and Substance Involvement Screening Test, MAQ = Medication Adherence Questionnaire, CARS = Childhood Autism Rating Scale, ABC = Aberrant Behaviour Checklist, SCQ = Social Communication Questionnaire, A-TAC = Autism-Tics ADHD and Comorbidities Inventory, MOSES = Monitoring of Side Effects Scale, CHU-9D = Child Health Utility-9D, WASI-II = Wechsler Abbreviated Scale of Intelligence-II, Vineland-3, SDSC = Sleep Disturbance Scale for Children, AQoL = Assessment of Quality of Life, FQoL = Family quality of Life, DASS = Depression Anxiety Stress Scale, APSI = Autism Parenting Stress Index, CASP = Child and Adolescent Scale of Participation, CSHQ = Children’s Sleep-Habit Questionnaire, CGI-I = Clinical Global Impression-Improvement scale, SRS = Social Responsiveness Scale, ADOS-2 = Autism Diagnostic Observation Schedule, VABS = Vineland Adaptive Behavior Scales, HSQ-AS = Home Situations Questionnaire-ASD, ABC-C FXS = Aberrant Behavior Checklist-Community Edition FXS, CaGI-S = Caregiver Global Impression-Severity, CaGI-C = Caregiver Global Impression-Change, HSQ-ASD = Home Situations Questionnaire-Autism Spectrum Disorder, VAMS = Visual Analogue Mood Scale, SSPS = Self-Statements during Public Speaking Scale, BSS = Bodily Symptoms Scale, SPIN = Social Phobia Inventory, CAARMS = Comprehensive Assessment of At-Risk Mental States, fMRI = functional magnetic resonance imaging, DMN = Default mode network, ATEC = Autism Treatment Evaluation Checklist. * Aran et al. (2021) and Schnapp et al. (2022) evaluated the same participants but different research questions

### Cannabinoid dosages used

Across the nine studies analyzed maximum CBD dosages ranged from 17.5 mg per day to 1000 mg per day. Only in one study [91] the maximum applied CBD dose per day was “only” 17.5 mg. In this cohort the mean age was also the lowest (mean age = 7.68 years). In all other trials maximum CBD doses ranged between 420 mg and 1000 mg per day. In only two trials MC was used and THC was co-administered (*n* = 1 trial, CBD: THC = 20:1; *n* = 1 trial, CBD: THC = 9:1). In those studies, maximum THC doses ranged from 1.95 mg to 21 mg per day. In total 305 individuals with mean ages ranging from 7.68 to 24.7 years of age were treated with CBD within the analyzed RCTs. Of those 195 received oral CBD via oil (*n* = 3 trials) and capsules (*n* = 4 trials). In one trial (*n* = 1 trial) 110 individuals received CBD topical, as transdermal gel [93] (See Tables 1 and 2).

### Safety– adverse effects– searching for a “safe dose”?

Across all analyzed studies no treatment related severe adverse events were reported. Three studies did not report any adverse events [94, 95, 97]. In the trial resulting in two publications by Aran et al. (2021) and Schnapp et al. (2022) three participants withdrew due to adverse events. They reported higher incidence of mild and moderate adverse events in the CTx group vs. the PLB group, without being statistically significant. Similar rates of adverse events were reported by da Silva Junior et al. (2024); Efron et al. (2020) and van Boxel et al. (2023). For the cohort receiving transdermal CBD gel about 54% of all patients experienced adverse events, with the main adverse symptom being site pain of the transdermal application [93] (See Table 5).

**Table 5:** Overview of cannabinoid naivity, concomitant medications, and adverse events

	Author	Dx	Cannabinoid naive?	Psychotropic medication	Somatic medication	Adverse Events (AE)	Serious adverse events (SAE)
1	Aran et al. (2021) [89]*	ASD	Not stated, but current or former CTx was exlusion criterion	Yes, ongoing, stable medication, e.g.: SGA, FGA, Anticonvusants, Stimulants, BZD, Melatonin, SSRI	Not stated	3 patients withdrew due to AEs, Mild AEs MC: 383, PC: 388, and PLB: 353 Moderate AEs MC: 80, PC: 78, and PLB: 57	No treatment-related SAE, *n* = 6 had an unrelated SE
2	Schnapp et al. (2022) [90]*	ASD	Not stated, but current or former CTx was exlusion criterion	Yes, ongoing, stable medication, e.g.: SGA, FGA, Anticonvusants, Stimulants, BZD, Melatonin, SSRI	Not stated	3 patients withdrew due to AEs. Mild AEs MC: 383, PC: 388, and PLB: 353 Moderate AEs MC: 80, PC: 78, and PLB: 57	No treatment-related SAE, *n* = 6 had an unrelated SE
3	da Silva Junior et al.(2024) [91]	ASD	Not stated, cannabis abstinence prior to trial	Approx. 50% of participants had psychotropic medication during trial, not further specified	Not stated	3 children in the CTx group had mild or moderate Aes	None reported
4	Efron et al. (2020) [92]	BP	Not stated, cannabis abstinence prior to trial	Yes, different combinations of: risperidone, methylphenidate, fluoxetine, guanfacin, valproate, melatonin	Yes: clonidine (*n* = 2)	Well tolerated, no dose reduction necessary More AEs were documented in CBD group compared to PLB. CBD 18; PLB: 8	Not reported
5	Berry-Kravis et al. (2022) [93]	BP	Not stated, cannabis abstinence prior to trial	Yes, ongoing, stable medication, not further specified	Not stated	54% of the 211 patients experienced adverse events All TEAEs were mild or moderate	No SAE reported
6	Wilson et al. (2019) [95]	PSY	Not necessarily; abstinence from cannabis, at least 96 h prior to trial	Not stated, anti-psychotic medication naive	Not stated	None reported	None reported
7	Appiah-Kusi et al. (2020) [94]	PSY	No	None	None	Not reported	Not reported
8	Van Boxel et al. (2023) [96]	PSY	No, cannabis use was tolerated, however CBD intake within one month prior to study was exclusion criterion	All patients had 1 stable antipsychotic medication	Not stated, but corticosteroids, carbamazepine or fluvoxamine were exclusion criteria	Mild AEs: CBD 34; PLB 16 Moderate AEs: CBD 4; PLB 4	None reported
9	Bergamaschi et al. (2011) [97]	ANX	No use of cannabis in the last 12 months prior to study particpation	None	None	None reported	None reported

Dx = Diagnosis/disorder type; AE = Adverse Event, SAE = Severe/Serious Adverse Event, CBD = Cannabidiol, BZD = Benzodiazepine, SGA = Second Generation Antipsychotic, FGA = First Generation Antipsychotic, SSRI = Selective Serotonin Reuptake Inhibitor, CTx = Cannbinoid Treatment, PLB = Placbeo, MC = Medicinal Cannabis, PC = Pharmaceutical Cannabis, CYP = Cytochrome, * Aran et al. (2021) and Schnapp et al. (2022) evaluated the same participants but different research questions

Generally, the applied cannabinoids and their respective dosages were reported to be well tolerated by the study participants, without any treatment related severe adverse events having been identified and stated. The applied dosages for children, adolescents and young adults were described above. The maximum applied dose of CBD per day was 20 mg/kg bodyweight (up to 1000 mg CBD per day as in Efron et al., (2020)). In the studies with 600 mg as daily CBD maximum in young adults we assumed a mean body weight of 70 kg and calculated an estimated dosage CBD of 8.57 mg/kg bodyweight as a conservative maximum dose for adults.

### Quantitative analysis and risk of bias analysis

Effect sizes for 13 primary outcomes (*n* = 13, 13 primary outcomes of 8 analyzed studies) were calculated and are displayed in Table 6; Fig. 1. For baseline data please see supplementary excel files (Supp_BL_Data.xlsx). Several outcomes required careful consideration for inclusion. The primary outcomes from Efron et al. were excluded due to insufficient statistical power (< 10 participants). Similarly, we could not include the Clinical Global Impression-Severity (CGI-S) outcomes from Aran et al., as their results were reported only as percentages of positive responders without clear baseline comparisons. Where appropriate, we differentiated between WP vs. PLB and PC vs. PLB comparisons. For the outcome of Berry-Kravis et al., we conducted separate effect size calculations for the total study population and a subgroup analysis focusing on Fragile-X patients with methylation levels exceeding 90%. Using a random-effects model, the overall pooled effect showed a modest positive effect (Hedges’ g = 0.308, 95% CI: 0.167, 0.448), though with substantial uncertainty in the estimate. Individual study effect sizes ranged from 0.116 (95% CI: −0.578, 0.809) to 0.997 (95% CI: 0.145, 1.849), with most studies showing wide confidence intervals (CI) that included zero (see Fig. 1).

**Table 6:** Primary outcome effect sizes

Study	Dx:	Outcome	Groups (*n*)	C’ d	Hedges’ g [95% CI]	I/E	Key Calculation	Notes
Aran et al. (2021) [89]*	ASD	HSQ-ASD	WP [40] vs. PLB [39]	0.413	0.409 [−0.037, 0.855]	I	d = (M₁-M₂)/SDpooled	Change scores, SD estimated from ranges
Aran et al. (2021) [89]*	ASD	HSQ-ASD	PC [42] vs. PLB [39]	0.122	0.120 [−0.316, 0.557]	I	d = (M₁-M₂)/SDpooled	Change scores, SD estimated from ranges
Aran et al. (2021) [89]*	ASD	CGI-S	WP/PC [45] vs. PLB [47]	-	-	E	-	Only baseline data available
Aran et al. (2021) [89]*	ASD	CGI-I	WP/PC [45] vs. PLB [47]	-	-	E	-	Only percentages of positive responses
Schnapp et al. (2022) [90]*	ASD	CSHQ	WP [44] vs. PLB [45]	0.147	0.146 [−0.270, 0.562]	I	d = (M₁-M₂)/SDpooled	Change scores with reported SDs
Schnapp et al. (2022) [90]*	ASD	CSHQ	PC [42] vs. PLB [45]	0.188	0.187 [−0.235, 0.608]	I	d = (M₁-M₂)/SDpooled	Change scores with reported SDs
da Silva Junior et al.(2024) [91]	ASD	ATEC T	WP [31] vs. PLB [29]	0.34	0.335 [−0.176, 0.845]	I	d = (M₁-M₂)/SDpooled	Post-only comparison
da Silva Junior et al.(2024) [91]	ASD	CARS	WP [31] vs. PLB [29]	0.499	0.492 [−0.022, 1.006]	I	d = (M₁-M₂)/SDpooled	Post-only comparison
Efron et al. (2020) [92]	BP	ABC	PC [3] vs. PLB [4]	-	-	E	-	Sample size too small (*n* < 10)
Efron et al. (2020) [92]	BP	DASS	PC [3] vs. PLB [4]	-	-	E	-	Sample size too small (*n* < 10)
Berry-Kravis et al. (2022) [93]	BP	ABC-CFXS SA	PC [110] vs. PLB [102]	0.137	0.137 [−0.627, 0.901]	I	d = LSMdiff/SDpooled	LSM change scores with SE
Berry-Kravis et al. (2022) [93]*	BP	ABC-CFXS SA	PC [76] vs. PLB [91]	0.719	0.716 [−0.127, 1.559]	I	d = LSMdiff/SDpooled	Methylation subgroup analysis
Wilson et al. (2019) [95]	PSY	RT	PC [16] vs. PLB [15]	0.126	0.123 [−0.583, 0.809]	I	d = (ΔM₁-ΔM₂)/SDpooled	Pre-post change comparison
Appiah-Kusi et al. (2020) [94]	PSY	Cortisol	PC [16] vs. PLB [16]	0.119	0.116 [−0.578, 0.809]	I	d = (ΔM₁-ΔM₂)/SDpooled	Pre-post change comparison
Appiah-Kusi et al. (2020) [94]	ANX	STAI	PC [16] vs. PLB [16]	0.31	0.302 [−0.395, 0.999]	I	d = (ΔM₁-ΔM₂)/SDpooled	Pre-post change comparison
Van Boxel et al. (2023) [96]	PSY	DMN	PC [16] vs. PLB [14]	0.8	0.778 [0.033, 1.523]	I	d = √(F×(n₁+n₂)/(n₁×n₂))	Calculated from time*treatment F-value
Bergamaschi et al. (2011) [97]	ANX	VAMS-Anxiety	PC [12] vs. PLB [12]	1.033	0.997 [0.145, 1.849]	I	d = √(F×(n₁+n₂)/(n₁×n₂))	Calculated from phases*group F-value

All effect sizes are positive, indicating improvement with cannabinoid treatment compared to placebo. Berry-Kravis* refers to subgroup analysis of participants with > 90% methylation. Confidence intervals in square brackets [lower limit, upper limit]. Abbreviations: Dx = Diagnosis/disorder type, ASD = Autism Spectrum Disorder, BP = Behavioral problems, PSY = Psychotic disorders; being at clinical high risk for psychosis, ANX = Anxiety disorder WP = Whole Plant extract, PC = Pure Cannabinoid, PLB = Placebo, n = sample size, g = Hedges’ g (corrected standardized mean difference), CI = Confidence Interval, I/E = Included/Excluded, LSM = Least Squares Mean, HSQ-ASD = Home Situations Questionnaire-Autism Spectrum Disorder, CSHQ = Children’s Sleep Habits Questionnaire, ATEC = Autism Treatment Evaluation Checklist, CARS = Childhood Autism Rating Scale, ABC-CFXS SA = Aberrant Behavior Checklist-Community Fragile X Syndrome Social Avoidance, RT = Reaction Time, STAI = State-Trait Anxiety Inventory, DMN = Default Mode Network, VAMS = Visual Analogue Mood Scale. Method Notes: Change scores indicate difference between pre- and post-treatment values, LSM change scores represent model-adjusted mean changes, Post-only refers to comparison of post-treatment values only, F-value interaction indicates effect sizes derived from time × treatment interaction F-statistics. Statistical Notes: All analyses conducted using random effects models

Subgroup analyses, accounting for the dependency structure, revealed comparable effects between WP preparations (g = 0.328, 95% CI: 0.083, 0.573, *n* = 4 outcomes from 3 independent trials) and pharmaceutical cannabinoids (g = 0.292, 95% CI: 0.069, 0.515, *n* = 9 outcomes from 6 independent trials). Duration analysis indicated that long-term interventions (g = 0.277, 95% CI: 0.132, 0.422, *n* = 8 outcomes from 3 independent trials) showed a smaller but more precise effect compared to short-term interventions (g = 0.416, 95% CI: −0.058, 0.891, *n* = 5 outcomes from 4 independent trials). Heterogeneity analyses, accounting for the dependency of outcomes from the same study population (particularly the Aran/Schnapp cohort), revealed low statistical heterogeneity (I² = 0%, τ² = 0, Q = 8.01, df = 12, *p* = 0.784). The multilevel meta-analysis, which explicitly modeled the dependency structure between outcomes from the same clinical trial, confirmed these findings with negligible variance components at both the study (σ² = 0.000) and outcome level (σ² = 0.000). This suggests that, despite the varying treatment durations, outcome measures, and the inclusion of multiple outcomes from the same study population, the results were remarkably consistent across studies. However, this low heterogeneity should be interpreted cautiously given the small number of independent trials included in the quantitative analysis (*n* = 7) and the wide confidence intervals observed in some subgroups.

Among clinical indications, primary outcomes for symptoms related to ASD demonstrated the most consistent evidence base (g = 0.264, 95% CI: 0.107, 0.421, *n* = 6 outcomes from 2 independent trials), while other subgroups showed considerably wider confidence intervals: BP (g = 0.398, 95% CI: −3.262, 4.059, *n* = 2 outcomes from 1 trial), psychotic disorder/CHR (g = 0.439, 95% CI: −3.723, 4.601, *n* = 2 outcomes from 2 trials), and anxiety (g = 0.415, 95% CI: −0.667, 1.497, *n* = 3 outcomes from 2 trials).

Generally, the analysis revealed complex relationships between age, cannabinoid dosing, and treatment effects. Age-stratified analysis showed that young adults (mean age 23.3 years, *n* = 5 outcomes from 117 participants) demonstrated higher effect sizes (g = 0.463, SD = 0.402) compared to pediatric populations (mean age 11.8 years, *n* = 8 outcomes from 357 participants; g = 0.318, SD = 0.212). However, this difference should be interpreted cautiously as age and dosing were confounded, with adult studies consistently using higher CBD doses (mean 600 mg/day) compared to pediatric studies (mean 339.4 mg/day).

Correlation analyses revealed no significant relationship between CBD dosage and effect size (ρ = −0.014, *p* = 0.963), and only a weak, non-significant positive correlation between age and effect size (ρ = 0.227, *p* = 0.457). Analysis by formulation type showed that oral capsule preparations, used exclusively in adult populations, demonstrated the highest mean effect size (g = 0.463, SD = 0.402), followed by topical gel applications used in pediatric populations (g = 0.426, SD = 0.409), while oil-based formulations showed more modest but more consistent effects (g = 0.282, SD = 0.153).

Risk of bias was performed using RoB 2-tool and *robvis* for visualization (see Fig. 2). Risk of bias for randomization processes and deviation from intended intervention were found to be low in all studies. For the publications (*n* = 5, four trials) on ASD and BP *some concerns* were found for missing outcome data, bias in measurement of the outcome and bias in selection of reported results. Due to *some concerns* in more than one domain of the RoB 2, these studies were assessed as overall *high risk of bias*. The three studies on psychotic disorders/CHR were rated as *low risk* for bias in all five domains (overall *low risk of bias*). The study on anxiety was rated with *some concerns* regarding bias in selection of the reported result (overall *moderate risk of bias*). However, these latter four trials were very short in duration ranging from one day/single application of CBD (*n* = 2), 7 days (*n* = 1), to 28 days (*n* = 1).

## Discussion

This systematic review and meta-analysis (for effect sizes of primary outcomes) provides an updated overview of RCTs on the therapeutic application of cannabinoids for children, adolescents, and young adults with psychiatric symptoms. We found that those studies qualifying for assessment (nine publications of eight clinical trials) studied the effects of cannabinoids on ASD, BP and psychotic disorders/CHR. The last systematic review by Rice et al. (2024) included literature published until April 2021 [18]. Then, the authors identified 5241 records through database searching and included 18 studies in their review. However, their study focused on human data (age range 0–18 years) and included one RCT, one open-label study, three observational trials, two case series and 11 case reports. In this systematic review we focused on updating the evidence by opting to include only RCTs. The study by Efron et al. (2020; *N* = 8) is the only RCT that was included both in the review conducted by Rice et al. (2024) and this systematic review. Furthermore, for this review we increased the age range to a mean age of 25 years for study populations that received CTx. The rationale to include trials with young adults aimed at covering the full transition age range typically referring to a period from late adolescence to early adulthood spanning from about 16 to 25 years in which most of psychiatric disorders emerge [9]. This includes schizophrenia with a peak age of onset of 20 to 25 years. Moreover, since brain maturation continues until the age of 25 [98], effects and side effects of medications/drugs may differ between age groups under and over 25 years.

### Qualitative synthesis

The efficacy of CTx for ASD remains uncertain based on the two RCTs that we assessed in this review. Aran et al. found that MC improved disruptive behavior, but not other outcomes like parenting stress [89]. Schnapp et al. reported no improvement in sleep disturbances with CTx compared to PLB [90]. In contrast, da Silva Junior et al. reported significant improvements in social interaction, anxiety, and psychomotor agitation following cannabinoid treatment. However, the generalizability of these findings is constrained by methodological limitations, including a relatively small sample size and the absence of baseline comparisons, as only post-treatment outcomes were reported. Moreover, the administered cannabinoid dosages were notably lower than those employed in comparable studies, further complicating direct comparisons with the other studies [91]. Overall, while there are some indications of efficacy, the evidence is mixed. The two studies on CTx for BP also showed mixed results. Efron et al. found that CBD may reduce severe BP in children with ID, though the small sample size limits the findings [92]. In a larger trial, Berry-Kravis et al. observed that ZYN002 CBD gel did not significantly reduce social avoidance in children with Fragile X Syndrome overall but did show benefits in a specific genetic subgroup [93]. The results of the three included studies of CTx for individuals at CHR or recent onset psychotic disorder are also mixed. Appiah-Kusi et al. found that CBD reduced stress-related cortisol reactivity and anxiety levels in CHR patients [94]. Wilson et al. observed that a single dose of CBD moderated abnormal brain activity in the insula, potentially reducing psychosis risk [95]. However, van Boxel et al. reported that while CBD increased DMN connectivity in patients with recent-onset psychosis, it had limited impact on other brain metabolites and reward processing [96]. Bergamaschi et al. found that a single 600 mg dose of CBD significantly reduced anxiety, cognitive impairment, and discomfort during a public speaking test in young adults with SAD, making their responses similar to HC. The PLB group experienced higher anxiety and discomfort, highlighting CBD’s potential efficacy in reducing anxiety [97].

### Quantitative synthesis

Our meta-analysis of effect sizes for primary outcomes presents preliminary evidence regarding CTx, with findings suggesting modest overall effectiveness but marked by substantial uncertainty in specific applications. The overall effect size (g = 0.308) should be interpreted with considerable caution given the heterogeneous nature of included studies and outcomes. While ASD studies emerged as the most consistent subgroup in our analysis, with a confidence interval not including zero, the modest effect size (g = 0.264) warrants careful interpretation. However, ASD studies also exhibited heterogeneity and the inclusion of da Silva Junior and colleagues’ study, which reported only post-treatment data without baseline comparisons, introduces potential confounding factors.

The comparable effects observed between WP and PC might suggest similar efficacy profiles, but the overlapping confidence intervals and uneven sample sizes preclude definitive conclusions about relative effectiveness. Similarly, while long-term interventions showed more precise estimates than short-term studies, this finding warrants cautious interpretation given the different sample sizes and methodological heterogeneity across studies. The relationship between age, dosage, and treatment response appears potentially non-linear. Adult studies, which exclusively used higher doses (600 mg/day) and oral capsule formulations, showed larger but more variable effects. However, this finding is likely confounded by the predominance of short-term and single-dose studies in the adult population, which may overestimate effect sizes compared to longer-term interventions. The analyzed primary outcomes of the pediatric studies employed more diverse dosing strategies and formulations, resulting in more consistent effects. A notable outlier in the pediatric group is the da Silva Junior study, which reported comparable effect sizes despite using substantially lower doses (17.5 mg CBD/day) compared to other pediatric studies (339.4 mg CBD/day mean dosage).

In total, several methodological considerations warrant attention when interpreting these quantitative findings. While our analysis included 13 primary outcomes from 8 studies (with Efron et al. excluded due to small sample size), the relatively small sample sizes in some studies and subgroups resulted in wide confidence intervals, particularly evident in the behavioral problems (2 primary outcomes) and psychotic disorder/CHR (2 primary outcomes) subgroups. The varying treatment durations (from single-dose to 12-week interventions) potentially affect the interpretation of treatment effects on primary outcomes. Although our heterogeneity analyses suggest consistent findings across different treatment durations (I² = 0%), the smaller effect sizes but narrower confidence intervals in long-term studies (g = 0.277, 95% CI: 0.132, 0.422, 8 primary outcomes) compared to short-term interventions (g = 0.416, 95% CI: −0.058, 0.891, 5 primary outcomes) suggest that treatment duration may influence both the magnitude and reliability of outcomes. The extreme width of confidence intervals in BP, CHR and anxiety subgroups underscores the substantial uncertainty in these areas. This imprecision, combined with the small number of studies per subgroup, suggests that current evidence is insufficient to draw meaningful conclusions about efficacy in these conditions. The varying levels of bias risk across included studies and the heterogeneous nature of outcome measures further complicate interpretation of the pooled effects.

### Adverse events and safety

Regarding adverse events all studies reported mild to moderate side effects of CTx, but no serious side effects (see Table 5). A maximum dosage of CBD 1000 mg/d and maximum co-administered doses of THC 21 mg/d were well tolerated. In recent safety analysis of CBD and CBD + THC in the population over 50 years of age showed that MC and PC are in general safe and acceptable in older adults [99]. Regarding adverse events another recent meta-analysis found that CBD is generally well-tolerated but is associated with increased risks of adverse events, particularly in childhood epilepsy studies where it may interact with other medications [100]. Another meta-analysis of CBD for epilepsy found associations with the development of several adverse events, such as somnolence, decreased appetite, diarrhea, and liver enzyme elevation. However, dosages ranged from 5 to 50 mg/kg bodyweight. The risk for serious adverse events was higher in the CBD-groups compared to PLB [101]. Seven trials analyzed in this review remained under the threshold of CBD 10 mg/kg bodyweight, one study with *n* = 4 participants treated with CBD had a maximum of 20 mg/kg body weight.

### Psychoactive drugs for minors?

While cannabinoids are different from psychedelics they share several similarities, particularly when considered for therapeutic use in the young [102, 103]. Both cannabinoids and psychedelics interact with the brain’s neurochemical systems, influencing mood, perception, and cognition. Furthermore, the ECS and serotonergic activity are closely linked and one system seems to modulate the other [104, 105]. While CBD also acts as an agonist at the 5HT_1A_ receptor, THC and other cannabinoids primarily interact with the ECS [106]. Classical psychedelics such as LSD, psilocybin and mescaline modulate serotonin receptors, particularly the 5-HT_2A_ receptor [107]. Both classes of substances are being investigated for their potential in treating severe psychiatric conditions, including anxiety, PTSD, and depression, especially when conventional treatments have failed [108]. Safety and neurodevelopmental concerns are paramount for both, especially when considering their use in minors. The developing brain’s vulnerability necessitates a cautious approach, with strict supervision in clinical settings to mitigate potential risks [109, 110].

Furthermore, both substance classes face significant legal and ethical challenges, as they are often heavily regulated [111]. This limits their availability and requires careful navigation of legal frameworks for their use in medical treatments, particularly in younger populations. The growing body of research into their therapeutic potential continues to shape our understanding of how these substances can be safely and effectively used.

Thus, cannabinoids, due to the existing scientific data on their therapeutic use and safety in minors, as well as the broader legal acceptance, could serve as a model for the therapeutic use of psychedelics in minors.

### Limitations and strengths of this review

To our knowledge this is the first review assessing only RCTs within the domain of child, adolescent and young adult-psychiatry and the application of cannabinoids with therapeutic intend. Due to significant heterogeneity in study designs, outcomes, and reporting methods, we restricted our quantitative analysis to primary outcomes only, as these represented the most robustly defined and reported endpoints across the studies. While we were able to calculate effect sizes for primary outcomes, the underlying heterogeneity in outcome measures, study populations, and intervention protocols suggests these results should be interpreted with considerable caution. This methodological challenge reflects a broader issue in cannabinoid research, where the rapid proliferation of studies has outpaced standardization of outcome measures and intervention protocols. The varying approaches to outcome measurement and reporting strongly limit the evidential strength of this review.

This systematic evaluation is especially timely given the increasing global trend toward cannabinoid legalization and the growing availability of cannabis-based products. From a clinical perspective, the rising interest from parents and caregivers seeking alternatives to conventional pharmacotherapy in child and adolescent psychiatry creates an urgent need for evidence-based guidance. Clinicians increasingly face the question, “Isn’t it possible to prescribe CBD for my young patient?”

To this date the meta-analysis by Black et al. (2019) remains the most thorough review, covering RCTs and observational studies on mental health conditions such as depression, anxiety, ADHD, Tourette syndrome, PTSD, and psychotic disorders in adults [19]. Their findings show limited evidence supporting the effectiveness of CBD or MC in treating these disorders. There was some low-quality evidence suggesting that pharmaceutical THC, with or without CBD, may help alleviate anxiety symptoms in people with other medical conditions like chronic non-cancer pain and multiple sclerosis [19]. A recent meta-analysis of animal studies found that pre-existing anxiety predicted larger effects of CBD on unconditioned anxiety. However tempting to conclude from these results that effective application in human patients may be possible, the quality of their evidence was still low [112]. Another recent critical narrative review on the potential benefits of CBD for psychiatric conditions in adult populations came to similar conclusions like previous findings– more evidence and mid-to-long-term data are needed [22].

### Implications for future research

Future research in this field would benefit from several key improvements. Larger, well-controlled trials are needed. Standardization of outcome measures across studies would facilitate more meaningful comparisons and meta-analyses. Direct comparisons between whole plant and pharmaceutical preparations, conducted with rigorous methodology and adequate sample sizes, would help clarify their relative benefits and risks. Additionally, longer-term follow-up studies with standardized protocols would provide much-needed evidence about sustained effectiveness and safety.

### Conclusion

Despite growing interest in society and the increased number of publications and ‘cannabinoid-supportive’ statements in the media, current evidence for efficacy of cannabinoids is still scarce. Our effect size analysis for primary outcomes revealed a modest positive overall effect and the use of cannabinoids was associated with few side effects and generally well tolerated. However, results from previously published reviews, meta-analyses, and our findings suggest there is still insufficient evidence to recommend prescribing cannabinoids for mental disorders in children, adolescents, and young adults. The growing interest in cannabinoid therapeutics, along with emerging trends in psychedelic medicine, underscores the crucial need for well-conducted, rigorous research with standardized protocols and outcome measures, particularly in young populations where neurodevelopmental considerations are paramount. Future research should prioritize large-scale, well-controlled trials with consistent methodologies to establish a more robust evidence base for clinical decision-making.

## Electronic supplementary material

Supplementary Material 1.

Supplementary Material 2.

Supplementary Material 3.

## Data Availability

No datasets were generated or analysed during the current study.
