# Supplementary material for: Cannabinoids for treating psychiatric disorders in youth: a systematic review of randomized controlled trials
Source: Child Adolesc Psychiatry Ment Health. 2024 Dec 18;18:158. doi: 10.1186/s13034-024-00846-5 (PMC11657296; doi:10.1186/s13034-024-00846-5)
Supplement: Supplementary file 1 — Supplementary Material 1. [file 13034_2024_846_MOESM1_ESM.docx]

# Required packages

library(meta)

library(metafor)

# 1. Change Score Studies (with positive values for improvement)

change_scores <- data.frame(

study = c("Aran WP", "Aran PC", "Schnapp WP", "Schnapp PC"),

n1 = c(40, 42, 44, 42),

n2 = c(39, 39, 45, 45),

m1 = c(-1.1, -0.7, -2.3, -2.9), # treatment change

m2 = c(-0.5, -0.5, -1.4, -1.4), # placebo change

sd1 = c(1.35, 1.73, 5.6, 9.2), # treatment SD

sd2 = c(1.55, 1.55, 6.6, 6.6) # placebo SD

)

# 2. Post-only Studies

post_only <- data.frame(

study = c("da Silva ATEC", "da Silva CARS"),

n1 = c(31, 31),

n2 = c(29, 29),

m1 = c(64.84, 33.47),

m2 = c(75.00, 37.83),

sd1 = c(26.82, 8.48),

sd2 = c(32.89, 9.02)

)

# 3. Berry-Kravis LSM Studies

berry_kravis <- data.frame(

study = c("Berry-Kravis", "Berry-Kravis >90%"),

n1 = c(110, 76),

n2 = c(102, 91),

diff = c(-0.39, -1.99), # LSM difference

se = c(0.39, 0.43) # Standard Error

)

# 4. Pre-Post Studies

prepost <- data.frame(

study = c("Wilson", "Appiah-Kusi Cort", "Appiah-Kusi STAI"),

n1 = c(16, 16, 16),

n2 = c(15, 16, 16),

pre1 = c(262.68, 397.15, 37.33),

post1 = c(248.00, 365.67, 44.31),

pre2 = c(251.93, 343.47, 38.07),

post2 = c(243.79, 297.47, 48.31),

sd_pre1 = c(58.6, 117.9, 8.66),

sd_post1 = c(49.72, 132.94, 11.11),

sd_pre2 = c(52.02, 121.02, 10.21),

sd_post2 = c(44.96, 115.07, 11.53)

)

# 5. F-statistic Studies

f_stat <- data.frame(

study = c("van Boxel", "Bergamaschi"),

n1 = c(16, 12),

n2 = c(14, 12),

f = c(4.775, 6.4)

)

# Effect Size Calculation Functions

# 1. For change scores

calc_change_es <- function(n1, n2, m1, m2, sd1, sd2) {

# Pooled SD

sd_pooled <- sqrt(((n1-1)*sd1^2 + (n2-1)*sd2^2)/(n1+n2-2))

# Cohen's d (absolute value since reduction = improvement)

d <- abs((m1 - m2)/sd_pooled)

# Standard Error

se <- sqrt((n1+n2)/(n1*n2) + d^2/(2*(n1+n2)))

return(c(d, se))

}

# 2. For post-only

calc_post_es <- function(n1, n2, m1, m2, sd1, sd2) {

# Pooled SD

sd_pooled <- sqrt(((n1-1)*sd1^2 + (n2-1)*sd2^2)/(n1+n2-2))

# Cohen's d

d <- abs((m1 - m2)/sd_pooled) # absolute value

# Standard Error

se <- sqrt((n1+n2)/(n1*n2) + d^2/(2*(n1+n2)))

return(c(d, se))

}

# 3. For Berry-Kravis (LSM)

calc_lsm_es <- function(n1, n2, diff, se) {

# Convert SE to SDpooled

sdpooled <- se/sqrt(1/n1 + 1/n2)

# Cohen's d

d <- abs(diff/sdpooled) # absolute value

return(c(d, se))

}

# For pre-post studies

calc_prepost_es <- function(n1, n2, pre1, post1, pre2, post2,

sd_pre1, sd_post1, sd_pre2, sd_post2,

r = 0.5) { # Added correlation parameter with default

# Calculate change scores

change_treat <- pre1 - post1

change_ctrl <- pre2 - post2

# SD of changes with specified correlation

sd_change1 <- sqrt(sd_pre1^2 + sd_post1^2 - 2*r*sd_pre1*sd_post1)

sd_change2 <- sqrt(sd_pre2^2 + sd_post2^2 - 2*r*sd_pre2*sd_post2)

# Pooled SD

sd_pooled <- sqrt(((n1-1)*sd_change1^2 + (n2-1)*sd_change2^2)/(n1+n2-2))

# Effect size

d <- abs((change_treat - change_ctrl)/sd_pooled)

# Standard Error

se <- sqrt((n1+n2)/(n1*n2) + d^2/(2*(n1+n2)))

return(c(d, se))

}

# 5. For F-statistics

calc_f_es <- function(n1, n2, f) {

# Cohen's d

d <- sqrt(f * (n1+n2)/(n1*n2))

# Standard Error

se <- sqrt((n1+n2)/(n1*n2) + d^2/(2*(n1+n2)))

return(c(d, se))

}

# Calculate all effect sizes

# Change scores

es_change <- t(mapply(calc_change_es,

change_scores$n1, change_scores$n2,

change_scores$m1, change_scores$m2,

change_scores$sd1, change_scores$sd2))

# Post-only

es_post <- t(mapply(calc_post_es,

post_only$n1, post_only$n2,

post_only$m1, post_only$m2,

post_only$sd1, post_only$sd2))

# Berry-Kravis

es_berry <- t(mapply(calc_lsm_es,

berry_kravis$n1, berry_kravis$n2,

berry_kravis$diff, berry_kravis$se))

# Pre-post

es_prepost <- t(mapply(calc_prepost_es,

prepost$n1, prepost$n2,

prepost$pre1, prepost$post1,

prepost$pre2, prepost$post2,

prepost$sd_pre1, prepost$sd_post1,

prepost$sd_pre2, prepost$sd_post2))

# F-statistics

es_f <- t(mapply(calc_f_es,

f_stat$n1, f_stat$n2,

f_stat$f))

# Combine all results

all_data <- data.frame(

study = c(as.character(change_scores$study),

as.character(post_only$study),

as.character(berry_kravis$study),

as.character(prepost$study),

as.character(f_stat$study)),

n1 = c(change_scores$n1, post_only$n1, berry_kravis$n1,

prepost$n1, f_stat$n1),

n2 = c(change_scores$n2, post_only$n2, berry_kravis$n2,

prepost$n2, f_stat$n2),

d = c(es_change[,1], es_post[,1], es_berry[,1],

es_prepost[,1], es_f[,1]),

se = c(es_change[,2], es_post[,2], es_berry[,2],

es_prepost[,2], es_f[,2])

)

# Calculate Hedges' g

all_data$g <- all_data$d * (1 - (3/(4*(all_data$n1 + all_data$n2 - 2) - 1)))

all_data$ci_lower <- all_data$g - 1.96*all_data$se

all_data$ci_upper <- all_data$g + 1.96*all_data$se

# Meta-analysis

meta_res <- metagen(TE = g,

seTE = se,

studlab = study,

data = all_data,

sm = "SMD",

method.tau = "REML",

hakn = TRUE,

prediction = TRUE,

comb.fixed = FALSE,

comb.random = TRUE)

# Create forest plot

pdf("forest_plot.pdf", width = 10, height = 8)

forest(meta_res,

sortvar = TE,

prediction = FALSE,

print.tau2 = TRUE,

leftlabs = c("Study", "g", "95% CI"),

label.e = "Favors Cannabinoid Treatment",

label.c = "Favors Placebo",

xlab = "Hedges' g",

smlab = "",

text.random = "Overall effect (random effects)",

col.diamond = "black",

col.diamond.lines = "black",

print.I2.ci = TRUE)

dev.off()

# Print formatted results

results_df <- data.frame(

Study = all_data$study,

N_Treatment = all_data$n1,

N_Control = all_data$n2,

Cohens_d = round(all_data$d, 3),

Hedges_g = round(all_data$g, 3),

CI_lower = round(all_data$ci_lower, 3),

CI_upper = round(all_data$ci_upper, 3)

)

print(results_df)

cat("\nHeterogeneity Statistics:\n")

cat("Q =", round(meta_res$Q, 2), "\n")

cat("df =", meta_res$df.Q, "\n")

cat("p =", round(meta_res$pval.Q, 3), "\n")

cat("I² =", round(meta_res$I2, 1), "%\n")

cat("τ² =", round(meta_res$tau2, 3), "\n")

cat("\nOverall Effect Size:\n")

cat("g =", round(meta_res$TE.random, 3),

"[", round(meta_res$lower.random, 3),

",", round(meta_res$upper.random, 3), "]\n")

#subgroup analyses

# Required packages

library(meta)

library(metafor)

# Add subgroup variables to the all_data dataframe

all_data$treatment_type <- c(

"WP", "PC", # Aran

"WP", "PC", # Schnapp

"WP", "WP", # da Silva

"PC", "PC", # Berry-Kravis

"PC", "PC", "PC", # Wilson, Appiah-Kusi

"PC", "PC" # van Boxel, Bergamaschi

)

all_data$duration <- c(

"long", "long", # Aran

"long", "long", # Schnapp

"long", "long", # da Silva

"long", "long", # Berry-Kravis

"short", "short", "short", # Wilson, Appiah-Kusi

"short", "short" # van Boxel, Bergamaschi

)

all_data$indication <- c(

"autism", "autism", # Aran

"autism", "autism", # Schnapp

"autism", "autism", # da Silva

"behavioral", "behavioral", # Berry-Kravis

"psychosis", "anxiety", "anxiety", # Wilson, Appiah-Kusi

"psychosis", "anxiety" # van Boxel, Bergamaschi

)

# Conduct subgroup analyses

# 1. By Treatment Type

meta_type <- metagen(TE = g,

seTE = se,

studlab = study,

data = all_data,

sm = "SMD",

byvar = treatment_type,

method.tau = "REML",

hakn = TRUE,

prediction = FALSE,

comb.fixed = FALSE,

comb.random = TRUE)

# 2. By Duration

meta_duration <- metagen(TE = g,

seTE = se,

studlab = study,

data = all_data,

sm = "SMD",

byvar = duration,

method.tau = "REML",

hakn = TRUE,

prediction = FALSE,

comb.fixed = FALSE,

comb.random = TRUE)

# 3. By Clinical Indication

meta_indication <- metagen(TE = g,

seTE = se,

studlab = study,

data = all_data,

sm = "SMD",

byvar = indication,

method.tau = "REML",

hakn = TRUE,

prediction = FALSE,

comb.fixed = FALSE,

comb.random = TRUE)

# Print results

cat("\nSubgroup Analysis by Treatment Type:\n")

print(summary(meta_type))

cat("\nSubgroup Analysis by Duration:\n")

print(summary(meta_duration))

cat("\nSubgroup Analysis by Clinical Indication:\n")

print(summary(meta_indication))

# Create forest plots for each subgroup analysis

# Treatment Type

pdf("forest_plot_treatment_type.pdf", width = 10, height = 8)

forest(meta_type,

sortvar = TE,

leftlabs = c("Study", "g", "95% CI"),

label.e = "Favors Cannabinoid Treatment",

label.c = "Favors Placebo",

xlab = "Hedges' g",

smlab = "Treatment Type",

text.random = "Subgroup effect",

col.diamond = "black",

col.diamond.lines = "black",

print.I2.ci = TRUE)

dev.off()

# Duration

pdf("forest_plot_duration.pdf", width = 10, height = 8)

forest(meta_duration,

sortvar = TE,

leftlabs = c("Study", "g", "95% CI"),

label.e = "Favors Cannabinoid Treatment",

label.c = "Favors Placebo",

xlab = "Hedges' g",

smlab = "Duration",

text.random = "Subgroup effect",

col.diamond = "black",

col.diamond.lines = "black",

print.I2.ci = TRUE)

dev.off()

# Clinical Indication

pdf("forest_plot_indication.pdf", width = 10, height = 8)

forest(meta_indication,

sortvar = TE,

leftlabs = c("Study", "g", "95% CI"),

label.e = "Favors Cannabinoid Treatment",

label.c = "Favors Placebo",

xlab = "Hedges' g",

smlab = "Clinical Indication",

text.random = "Subgroup effect",

col.diamond = "black",

col.diamond.lines = "black",

print.I2.ci = TRUE)

dev.off()

# Create summary table for subgroup analyses

subgroup_table <- data.frame(

Subgroup = character(),

Effect_Size = numeric(),

CI_Lower = numeric(),

CI_Upper = numeric(),

Number_Studies = numeric(),

stringsAsFactors = FALSE

)

# Function to extract subgroup data

extract_subgroup_data <- function(meta_obj) {

results <- summary(meta_obj)

data.frame(

Subgroup = results$bylevs,

Effect_Size = round(results$TE.random.w, 3),

CI_Lower = round(results$lower.random.w, 3),

CI_Upper = round(results$upper.random.w, 3),

Number_Studies = results$k.w

)

}

# Get data for each analysis type

type_data <- extract_subgroup_data(meta_type)

duration_data <- extract_subgroup_data(meta_duration)

indication_data <- extract_subgroup_data(meta_indication)

# Combine all data

all_subgroups <- rbind(

type_data,

duration_data,

indication_data

)

# Print nicely formatted table

print(all_subgroups)

# Or save to CSV

write.csv(all_subgroups, "subgroup_analysis_summary.csv", row.names = FALSE)

# Required packages

library(metafor)

# 1. Create outcome identifier

all_data$outcome <- 1:nrow(all_data) # unique identifier for each outcome

all_data$study_id <- as.numeric(factor(all_data$study)) # numeric study identifier

# 2. Multilevel meta-analysis

meta_multi <- rma.mv(yi = g,

V = se^2,

random = ~ 1 | study_id/outcome,

data = all_data,

method = "REML")

# 3. Alternative heterogeneity analysis

meta_alt <- rma(yi = g,

sei = se,

data = all_data,

method = "REML",

test = "knha")

# 4. Print results

summary(meta_multi)

summary(meta_alt)

# 5. Calculate I² for multilevel model

W <- diag(1/all_data$se^2)

X <- model.matrix(meta_multi)

P <- W - W %*% X %*% solve(t(X) %*% W %*% X) %*% t(X) %*% W

I2_multi <- 100 * sum(meta_multi$sigma2) / (sum(meta_multi$sigma2) + (meta_multi$k-meta_multi$p)/sum(diag(P)))

# 6. Create results table

results_table <- data.frame(

Model = c("Standard Random-Effects", "Multilevel"),

tau2 = c(meta_alt$tau2, sum(meta_multi$sigma2)),

I2 = c(meta_alt$I2, I2_multi),

Q = c(meta_alt$QE, meta_multi$QE),

p_value = c(meta_alt$QEp, meta_multi$QEp)

)

print(results_table)

# Function to run sensitivity analysis

calculate_sensitivity <- function(r_value) {

# Calculate effect sizes for pre-post studies

prepost_sens <- t(mapply(calc_prepost_es,

prepost$n1, prepost$n2,

prepost$pre1, prepost$post1,

prepost$pre2, prepost$post2,

prepost$sd_pre1, prepost$sd_post1,

prepost$sd_pre2, prepost$sd_post2,

MoreArgs = list(r = r_value))) # Specify r as a constant

# Combine with other unchanged effect sizes

d_sens <- c(es_change[,1], es_post[,1], es_berry[,1],

prepost_sens[,1], es_f[,1])

se_sens <- c(es_change[,2], es_post[,2], es_berry[,2],

prepost_sens[,2], es_f[,2])

# Run meta-analysis

meta_sens <- rma(yi = d_sens,

sei = se_sens,

method = "REML",

test = "knha")

return(meta_sens)

}

# Run sensitivity analyses

sens_0.3 <- calculate_sensitivity(0.3)

sens_0.7 <- calculate_sensitivity(0.7)

# Create results table

heterogeneity_results <- data.frame(

Method = c("Standard Random-Effects", "Multilevel",

"Sensitivity r=0.3", "Sensitivity r=0.7"),

tau2 = round(c(meta_alt$tau2, sum(meta_multi$sigma2),

sens_0.3$tau2, sens_0.7$tau2), 3),

I2 = round(c(meta_alt$I2, I2_multi,

sens_0.3$I2, sens_0.7$I2), 1),

Q = round(c(meta_alt$QE, meta_multi$QE,

sens_0.3$QE, sens_0.7$QE), 2),

p_value = round(c(meta_alt$QEp, meta_multi$QEp,

sens_0.3$QEp, sens_0.7$QEp), 3)

)

# Print results

print(heterogeneity_results)

# Required packages

library(ggplot2)

library(dplyr)

library(tidyr)

# First calculate heterogeneity statistics

manual_het <- function(yi, sei) {

# Calculate weights

wi <- 1/sei^2

# Calculate weighted mean

M <- sum(wi * yi) / sum(wi)

# Calculate Q statistic

Q <- sum(wi * (yi - M)^2)

# Calculate df

df <- length(yi) - 1

# Calculate I²

I2 <- ifelse(Q > df,

100 * (Q - df)/Q,

0)

# Calculate τ²

C <- sum(wi) - sum(wi^2)/sum(wi)

tau2 <- max(0, (Q - df)/C)

return(list(

Q = Q,

df = df,

M = M,

I2 = I2,

tau2 = tau2,

p_value = 1 - pchisq(Q, df)

))

}

# Calculate heterogeneity statistics

het_stats <- manual_het(yi = all_data$g, sei = all_data$se)

# Now create visualizations

# 1. Effect sizes with confidence intervals

p1 <- ggplot(all_data, aes(y = reorder(study, g), x = g)) +

geom_point(aes(size = 1/se^2)) +

geom_errorbarh(aes(xmin = g - 1.96*se, xmax = g + 1.96*se)) +

geom_vline(xintercept = het_stats$M, linetype = "dashed", color = "red") +

theme_minimal() +

labs(x = "Hedges' g", y = "Study",

title = "Effect Sizes with Precision Weighting",

subtitle = "Point size indicates study precision (1/SE²)")

# 2. Effect sizes by indication

p2 <- ggplot(all_data, aes(x = indication, y = g, color = indication)) +

geom_point(position = position_jitter(width = 0.2)) +

geom_boxplot(alpha = 0.5) +

theme_minimal() +

labs(title = "Effect Sizes by Clinical Indication",

x = "Clinical Indication",

y = "Hedges' g")

# 3. Sample size vs effect size

p3 <- ggplot(all_data, aes(x = n1 + n2, y = g)) +

geom_point() +

geom_smooth(method = "lm", se = TRUE) +

theme_minimal() +

labs(x = "Total Sample Size", y = "Hedges' g",

title = "Effect Size vs Sample Size")

# Create tables

# Subset analysis

subset_analysis <- all_data %>%

group_by(indication) %>%

summarize(

n_studies = n(),

mean_g = mean(g),

sd_g = sd(g),

median_g = median(g),

mean_se = mean(se),

total_n = sum(n1 + n2)

)

# Contribution to heterogeneity

contribution <- data.frame(

study = all_data$study,

effect_size = all_data$g,

weight = 1/all_data$se^2,

deviation = abs(all_data$g - het_stats$M),

contribution = (all_data$g - het_stats$M)^2 * (1/all_data$se^2)

) %>%

arrange(desc(contribution))

# Save plots

pdf("heterogeneity_analysis_plots.pdf", width = 10, height = 15)

print(p1)

print(p2)

print(p3)

dev.off()

# Print results

cat("\nHeterogeneity Statistics:\n")

cat("Q =", round(het_stats$Q, 2), "\n")

cat("df =", het_stats$df, "\n")

cat("M =", round(het_stats$M, 3), "\n")

cat("I² =", round(het_stats$I2, 1), "%\n")

cat("τ² =", round(het_stats$tau2, 3), "\n")

cat("p =", round(het_stats$p_value, 3), "\n")

cat("\nSubset Analysis by Indication:\n")

print(subset_analysis)

cat("\nTop Contributors to Heterogeneity:\n")

print(head(contribution))

# Write results to CSV

write.csv(subset_analysis, "subset_analysis.csv", row.names = FALSE)

write.csv(contribution, "heterogeneity_contribution.csv", row.names = FALSE)

# Load required packages

library(tidyverse)

library(correlation)

library(ggplot2)

library(knitr)

# Create updated dataset

cannabinoid_data <- data.frame(

study = c("Aran WP", "Aran PC", "Schnapp WP", "Schnapp PC",

"da Silva ATEC", "da Silva CARS",

"Berry-Kravis", "Berry-Kravis >90%",

"Wilson", "Appiah-Kusi Cort", "Appiah-Kusi STAI",

"van Boxel", "Bergamaschi"),

hedges_g = c(0.409, 0.120, 0.146, 0.187,

0.335, 0.492,

0.137, 0.716,

0.123, 0.116, 0.302,

0.778, 0.997),

formulation = c("oil", "oil", "oil", "oil",

"oil", "oil",

"gel", "gel",

"capsule", "capsule", "capsule",

"capsule", "capsule"),

application = c("oral", "oral", "oral", "oral",

"oral", "oral",

"topical", "topical",

"oral", "oral", "oral",

"oral", "oral"),

cbd_dose = c(420, 420, 420, 420,

17.5, 17.5,

500, 500,

600, 600, 600,

600, 600),

thc_dose = c(21, 21, 21, 21,

1.95, 1.95,

0, 0,

0, 0, 0,

0, 0),

type = c("WP", "PC", "WP", "PC",

"WP", "WP",

"PC", "PC",

"PC", "PC", "PC",

"PC", "PC"),

mean_age = c(11.8, 11.8, 11.8, 11.8,

13.9, 13.9,

9.7, 9.7,

22.7, 22.33, 22.33,

24.7, 24.6),

age_group = c("pediatric", "pediatric", "pediatric", "pediatric",

"pediatric", "pediatric",

"pediatric", "pediatric",

"adult", "adult", "adult",

"adult", "adult")

)

# Calculate correlations

cbd_correlation <- cor.test(cannabinoid_data$cbd_dose,

cannabinoid_data$hedges_g,

method = "spearman")

age_correlation <- cor.test(cannabinoid_data$mean_age,

cannabinoid_data$hedges_g,

method = "spearman")

# Analysis by age group

age_summary <- cannabinoid_data %>%

group_by(age_group) %>%

summarise(

mean_g = mean(hedges_g),

sd_g = sd(hedges_g),

n = n(),

mean_age = mean(mean_age),

mean_cbd = mean(cbd_dose)

)

# Analysis by formulation and age

formulation_age_summary <- cannabinoid_data %>%

group_by(formulation) %>%

summarise(

mean_g = mean(hedges_g),

sd_g = sd(hedges_g),

n = n(),

mean_age = mean(mean_age),

mean_cbd = mean(cbd_dose)

)

# Create visualization for age and effect size

ggplot(cannabinoid_data, aes(x = mean_age, y = hedges_g, color = formulation)) +

geom_point(size = 3) +

geom_smooth(method = "lm", se = TRUE) +

theme_minimal() +

labs(x = "Mean Age (years)",

y = "Hedges' g",

title = "Relationship between Age and Effect Size",

color = "Formulation Type")

# Print results

print("Correlation between CBD dose and effect size:")

print(cbd_correlation)

print("\nCorrelation between age and effect size:")

print(age_correlation)

print("\nEffect sizes by age group:")

print(age_summary)

print("\nEffect sizes by formulation including age:")

print(formulation_age_summary)
