# Supplementary material for: Cannabinoids for treating psychiatric disorders in youth: a systematic review of randomized controlled trials
Source: Child Adolesc Psychiatry Ment Health. 2024 Dec 18;18:158. doi: 10.1186/s13034-024-00846-5 (PMC11657296; doi:10.1186/s13034-024-00846-5)
Supplement: Supplementary file 3 — Supplementary Material 3. [file 13034_2024_846_MOESM3_ESM.docx]

**Appendix I**

Abbreviations for the article “Cannabinoids for Treating Psychiatric Disorders in Youth: A Sys-tematic Review of Randomized Controlled Trials” by Köck et al.

**Substances investigated, study terminology**

CBMP = Cannabis-Based Medicinal Product
CBD = Cannabidiol
CTx = Cannabinoid Treatment
HC = Healthy Control
MC = Medicinal Cannabis
PC = Pharmaceutical Cannabis
PLB = Placebo
RCT = Randomized Controlled Trial
THC = Tetrahydrocannabinol

**Disorder types**

ADHD = Attention Deficit and Hyperactivity Disorder
ASD = Autism Spectrum Disorder
BP = Behavioral Problems
CHR = Clinical High Risk for Psychosis
ID = Intellectual Disability
NDD = Neurodevelopmental Disorders
SAD = Social Anxiety Disorder
SBP = Severe Behavioral Problems

**Questionnaires**

ABC = Aberrant Behaviour Checklist
ABC-C FXS = Aberrant Behavior Checklist-Community Edition FXS
ADOS-2 = Autism Diagnostic Observation Schedule
APIS = Autism Parenting Stress Index
AQoL = Assessment of Quality of Life
ATEC = Autism Treatment Evaluation Checklist
A-TAC = Autism-Tics ADHD and Comorbidities Inventory
BACS = Brief Assessment of Cognition in Schizophrenia
BSS = Bodily Symptoms Scale
CAARMS = Comprehensive Assessment of At-Risk Mental States
CaGI-C = Caregiver Global Impression-Change
CaGI-S = Caregiver Global Impression-Severity
CARS = Childhood Autism Rating Scale
CASP = Child and Adolescent Scale of Participation
CHU-9D = Child Health Utility-9D
CGI = Clinical Global Impression
CGI-I = Clinical Global Impression-Improvement scale
CSHQ = Children's Sleep-Habit Questionnaire
DASS = Depression Anxiety Stress Scale
FQoL = Family Quality of Life
GAF = Global Assessment of Functioning scale
HAM-D = Hamilton Depression Scale
HSQ-AS = Home Situations Questionnaire-ASD
HSQ-ASD = Home Situations Questionnaire-Autism Spectrum Disorder
MAQ = Medication Adherence Questionnaire
MOSES = Monitoring of Side Effects Scale
PANSS = Positive and Negative Syndrome Scale
SCQ = Social Communication Questionnaire
SDSC = Sleep Disturbance Scale for Children
SPIN = Social Phobia Inventory
SOFAS = Social and Occupational Functioning Assessment Scale
SRS = Social Responsiveness Scale
SSPS = Self-Statements during Public Speaking Scale
VABS = Vineland Adaptive Behavior Scales
VAMS = Visual Analogue Mood Scale
Vineland-3 = Vineland Adaptive Behavior Scales – Third Edition
WASI-II = Wechsler Abbreviated Scale of Intelligence-II
WHO Assist 3.0 = World Health Organization Alcohol, Smoking, and Substance Involvement Screening Test
YMRS = Young Mania Rating Scale

fMRI = functional magnetic resonance imaging

DMN = Default mode network

ACC = Anterior cingulate cortex

**Statistical terms**

d = Cohen's d (standardized mean difference effect size)

g = Hedges' g (bias-corrected standardized mean difference effect size)

CI = Confidence Interval

SD = Standard Deviation

SE = Standard Error

f = F-statistic (ratio of variances between groups)

I² = I-squared (percentage of variation across studies due to heterogeneity)

τ² = Tau-squared (estimate of between-study variance)

Q = Cochran's Q (test statistic for heterogeneity) p = probability value (statistical significance)

ρ (rho) = Spearman's rank correlation coefficient

df = degrees of freedom

N = total sample size

n = subsample size / primary outcomes

LSM = Least Square Means

REML = Restricted Maximum Likelihood Estimation
